# Repurposing NGO data for better research outcomes: a scoping review of the use and secondary analysis of NGO data in health policy and systems research

**DOI:** 10.1186/s12961-020-00577-x

**Published:** 2020-06-08

**Authors:** Sarah C. Masefield, Alice Megaw, Matt Barlow, Piran C. L. White, Henrice Altink, Jean Grugel

**Affiliations:** 1 Department of Health Sciences, University of York, York, YO10 5DD United Kingdom; 2 Interdisciplinary Global Development Centre, University of York, York, YO10 5DD United Kingdom; 3 Department of Politics, University of York, York, YO10 5DD United Kingdom; 4 Department of Environment and Geography, University of York, York, YO10 5NG United Kingdom; 5 Department of History, University of York, York, YO10 5NH United Kingdom

**Keywords:** Non-government organisations, Health policy and systems research, Developing countries, Secondary data analysis, Marginalised groups, Sustainable development goals

## Abstract

**Background:**

Non-governmental organisations (NGOs) collect and generate vast amounts of potentially rich data, most of which are not used for research purposes. Secondary analysis of NGO data (their use and analysis in a study for which they were not originally collected) presents an important but largely unrealised opportunity to provide new research insights in critical areas, including the evaluation of health policy and programmes.

**Methods:**

A scoping review of the published literature was performed to identify the extent to which secondary analysis of NGO data has been used in health policy and systems research (HPSR). A tiered analytical approach provided a comprehensive overview and descriptive analyses of the studies that (1) used data produced or collected by or about NGOs; (2) performed secondary analysis of the NGO data (beyond the use of an NGO report as a supporting reference); and (3) analysed NGO-collected clinical data.

**Results:**

Of the 156 studies that performed secondary analysis of NGO-produced or collected data, 64% (*n* = 100) used NGO-produced reports (mostly to a limited extent, as a contextual reference or to critique NGO activities) and 8% (*n* = 13) analysed NGO-collected clinical data. Of these studies, 55% (*n* = 86) investigated service delivery research topics and 48% (*n* = 51) were undertaken in developing countries and 17% (*n* = 27) in both developing and developed countries. NGOs were authors or co-authors of 26% of the studies. NGO-collected clinical data enabled HPSR within marginalised groups (e.g. migrants, people in conflict-affected areas), albeit with some limitations such as inconsistent and missing data.

**Conclusion:**

We found evidence that NGO-collected and produced data are most commonly perceived as a source of supporting evidence for HPSR and not as primary source data. However, these data can facilitate research in under-researched marginalised groups and in contexts that are hard to reach by academics such as conflict-affected areas. NGO–academic collaboration could help address issues of NGO data quality to facilitate their more widespread use in research. The use of NGO data use could enable relevant and timely research in the areas of programme evaluation and health policy and advocacy to improve health and reduce health inequalities, especially in marginalised groups and developing countries.

## Background

The lower estimate of the number of non-governmental organisations (NGOs; non-profit groups formed voluntarily) in the world is 1 million, but there may be as many as 10.3 million (based on the number of registrations of .org and .ngo domain names) [1, 2]. An estimated 35,000 are large, established NGOs and many operate in the health sector; in the year 2000, there were over 2000 international health NGOs and this number is expected to have increased since [3, 4]. These NGOs deliver, monitor and advocate for health services and equitable healthcare at the community, national and international levels [4, 5]. To a lesser extent, they are engaged in performing and disseminating research [1, 6, 7].

Health policy and systems research (HPSR) is a multidisciplinary field of research conducted to inform and influence policies and systems to improve health outcomes for all [8, 9]. Within the context of HPSR, NGOs necessarily produce data on the services and programmes they deliver, collect data on the (often marginalised and hard to reach by researchers) recipient populations and the health conditions being treated [10, 11]. These data can be patient records for populations who do not access national healthcare, administrative data on the number of drugs dispensed or days that patients spent in NGO-run healthcare facilities, data on community responses to health crises (e.g. in the aftermath of extreme weather events), or reviews of health policy [12]. Although NGOs are only rarely collecting data for the purposes of research, the records and data held by them are a vast (and largely untapped) source of potentially rich data [6].

NGO-collected data are especially valuable for research in developing countries, on populations under-served by the national health system, and where there may be a data gap due to inadequate national data collection and monitoring infrastructure [6, 13, 14]. The analysis of NGO data presents an opportunity for researchers to conduct relevant, timely and relatively cheap secondary research that has the potential to improve health outcomes [6, 15–17]. However, there is a dual problem of these data being ignored by researchers and not made available by NGOs for secondary analysis [6, 18, 19]. Although some examples of NGO–academic collaboration and open access repositories for NGO data exist [11, 20, 21], at other times, researchers will have to approach NGOs to request access to data about them or collected by them [22, 23].

HPSR is led largely by questions from ‘the field’ rather than being theory driven but conceptual frameworks are used to describe and analyse the health systems studied [24]. For example, the WHO framework of the six health system building blocks required to improve health and health equity uses the categories of (1) service delivery; (2) healthcare workforce; (3) information; (4) medical products, vaccines and technologies; (5) financing; and (6) leadership and governance [25]. The framework is widely used in HPSR, particularly in developing country contexts, as it helps locate, describe and classify health system constraints, where investment is needed, and how change can be monitored [24, 26–30].

Although the WHO framework has received some criticism (e.g. a lack of inter-connectedness between the blocks and a failure to place healthcare recipients at the centre of the health system), it remains widely used in HPSR as it is founded on the human right of the highest standard of physical and mental health and reinforces improved implementation (universal access to efficient health services) as the research outcome [25, 29, 31]. NGO data, which are, by their nature, collected in ‘the field’, therefore have considerable potential to inform and improve research into the questions asked in HPSR [7, 32]. We used the WHO building blocks framework to assess the appropriateness of studies for our scoping review as it would enable a broad overview of the different areas of HPSR in which NGO data are used and we expected most of the studies in our review to use data from developing countries [33].

Secondary analysis is the analysis of qualitative or quantitative data not produced or collected for the study in which they are later used [34, 35]. The secondary analysis of data collected, generated or about health NGOs can provide valuable insights into healthcare practice, highlight discontinuity between policy and practice, demonstrate inequitable access to healthcare, and show changes over time [18, 36]. For example, data from the patient records of NGO-run health facilities can be compared with those of government-run facilities [37]. These comparisons can highlight differences in the health and services available to populations with different sociodemographic, health or other characteristics [37, 38]. However, secondary analysis of NGO data is used infrequently in academic research in general and in HPSR in particular [10, 15, 39]. To our knowledge, there have been no previous studies on what HPSR has been conducted through secondary analysis of NGO data with a view to making recommendations to prevent the ongoing underuse of these valuable sources of information.

Health NGOs act in the public arena to improve the health and represent the health-related interests of specific groups of people or of society as a whole. Their most common undertaking is health service delivery. They are frequently commissioned by public authorities to provide services or they identify and meet the service needs of a particular and often marginalised population group (we use the term ‘marginalised groups’ to include vulnerable and hard-to-reach population groups), whilst the public health system provides more generic services [40]. In many countries in the global south, whilst universal health coverage may be the stated aim of health systems [41], in locations or disease areas with low national health system coverage, private and NGO providers frequently step in. For example, in Malawi, the government provides 62% of health services, with 37% being provided by the NGO Christian Health Association of Malawi [42, 43]. In areas affected by conflict or natural disasters, NGOs often operate as an emergency health system until there is sufficient stability for public services to be reinstated or NGO services are scaled up through NGO–public/private collaboration [44]. NGO data can therefore sometimes be the only data available in some settings or for certain population groups [44, 45].

NGOs can also perform an essential monitoring function – assessing the scale of healthcare needs and identifying the disease and related healthcare priorities and issues in accessing health services [26]. This can be especially important for settings where the public healthcare system has collapsed or provision is reduced (often affecting the most marginalised communities), whether in everyday settings in much of the global south or during disasters or crises.

As NGOs work with the patient groups, they are also increasingly involved in advocacy to influence health policy and education to ensure the capacity and sensitivity of health workforces and systems to address the needs of the community [4, 46, 47]. For example, NGOs perform independent reviews of services or reports on humanitarian crises. As such, NGOs have an opinion-forming role, sometimes formalised in the guise of think tanks, in addition to the roles of service delivery and monitoring. These varied operations illustrate NGOs’ diverse potential engagement with HPSR as the end-user (the implementer) and/or funder, data source, author or collaborator. As such, they are a key stakeholder in HPSR – they can inform the HPSR research agenda, benefit from HPSR research, and disseminate HPSR findings to the study populations and other non-academic stakeholders [48, 49].

Despite the obvious potential mutual benefits and shared aim of improving health outcomes, challenges to the use of NGO data by academics and NGO–academic collaboration remain and are well documented [7, 11, 15, 50]. Concerns include time and funding for collaboration, lack of methodological rigour and poor data quality [18, 51, 52]. For example, the timeframes of academics and NGOs can differ as the NGOs often prioritise developing trust and collaboration with the patient group whilst academics may be more concerned with data collection over a short time period [53]. Another example is the pragmatic data collection that NGOs tend to employ, rather than being concept or research-question driven. This can result in data that are inconsistent or partial, with data sometimes collected in differing samples (e.g. locations), over different timeframes and not always available in easily accessible formats [36, 54]. This causes practical difficulties of aggregation and uncertainties in measures and interpretation [6]. Academic concerns about secondary analysis of qualitative data, such as interviews or focus groups more generally, and not just qualitative data collected by NGOs include the inability to verify the source and key characteristics, difficulties assessing and addressing any bias in the data collection process, e.g. via additional data collection, and the collection of data to fit their research questions [55]. Despite these challenges, the volume, access and often depth as well as the immediacy of data generated by NGOs represent significant untapped potential for secondary analysis, especially for population groups where there is no other source of data [48].

## Methods

A scoping review of existing published literature was conducted following the framework initially outlined by Arksey and O’Malley [56]. Scoping reviews aim to examine the extent, range and nature of research activity by ‘charting’ the key concepts underlying the research area and the main sources and types of evidence available. They are valuable for gaining a rapid understanding of areas that are complex and/or have not been reviewed comprehensively before.

We used the five core stages of the methodological framework, namely (1) identify the research question; (2) identify relevant studies; (3) select the studies; (4) chart the data; and (5) collate, summarise and report the results. It was outside the scope of this review to perform the optional ‘sixth stage’ consultation exercise to validate the findings of the review. As a scoping review, breadth rather than quality of the studies was prioritised. A quality appraisal was not performed [56].

### Stage 1: identifying the research question

The primary objective of this review was to summarise and critically appraise the extent to which NGO data have been used in HPSR in relation to the types of NGO data used, the ways in which they have been used and areas of HPSR to which they have been applied, and to identify opportunities for greater use in future research via secondary analysis. We seek to show how these data are being used in the HPSR context and highlight their potential for health system development, particularly in developing countries [57].

### Stage 2: identifying relevant studies

We performed a systematic search of papers published between January 2010 and February 2019 in the databases Web of Science, Scopus, Medline (OVID), and Health Management Information Consortium (HMIC). A post-hoc search was performed in Embase (Ovid) (Table 1).

**Table 1:** The literature search strategies used in the different databases

Web of Science: Topic = (“health” AND (“non-governmental” OR NGO* OR “community organization*” OR “charity” OR “community group” OR “civil society organization*”) AND (“governance” OR “system” OR “delivery”)) Timespan: 2010–2019	
Scopus: Title-Abstract-Keywords (“health” AND (“non-governmental” OR NGO* OR “community organization*” OR “charity” OR “community group” OR “civil society organization*”) AND (“governance” OR “system” OR “delivery”)) AND PUBYEAR > 2009	
Medline: Title-Abstract-Keywords = (health AND (non-governmental OR NGO* OR community organization* OR charity OR community group OR civil society organization*) AND (governance OR system OR delivery) limit to yr = “2010”)	
Health Management Information Consortium: Title-Abstract-Keywords = (“health” AND (“non-governmental” OR NGO* OR “community organization*” OR “charity” OR “community group” OR “civil society organization*”) AND (“governance” OR “system” OR “delivery”) limit to yr = “2010”)	
Embase: Title-Abstract-Keyword = (“health” or “medicine”) AND (“non-governmental” OR NGO* OR “community organization*” OR “charity” OR “charities” OR “community group*” OR “civil society organization*”) AND (“governance” OR “system*” OR “delivery” OR “policy” OR “policies”) limit to yr = “2010”	

Initial searches were carried out using Web of Science, Scopus, Medline and Health Management Information Consortium. A post-hoc search was performed in Embase in response to concerns that the initial strategy may have missed some studies, particularly on health policy

Searching interdisciplinary databases (Web of Science, Scopus) as well as those with a health focus (Medline, HMIC) ensured a comprehensive and inclusive approach as relevant papers were expected in journals for the social sciences, particularly global development, as well as health research. HMIC covers the areas of health service policy, management and administration, and public health. It contains information from DH Data (produced by the United Kingdom Department of Health) and the King’s Fund Information and Library Service database but is not limited to United Kingdom-only research.

A broad search strategy was used to identify records with an NGO key term (including community organisation/group, charity, civil society organisation) and the terms health system, delivery or governance in the title, abstract or key terms. The key terms used were informed by the authors’ knowledge of NGOs, development and health research in both high- and low-income countries. To increase the sensitivity of the searches, the key terms were mapped to subject headings, where possible. The date range was restricted to manage the large number of records retrieved by the strategy and to meet the research objective for the contemporary HPSR arena.

Following the screening process (Stage 3 below), a further post-hoc search was performed for the same period in the database Embase (Ovid), which includes some medical and related journals not indexed in Medline (Ovid). This aimed to increase the number of studies available for the review in response to concerns that the initial strategy may have missed some studies, particularly on health policy, or which used the key term ‘medicine’ rather than ‘health’.

### Stage 3: study selection

Titles and abstracts, followed by the full text of potentially included articles, were screened according to the inclusion criteria by at least two reviewers (SM, AM, MB; only SM reviewed the citations identified via the post-hoc Embase search). The article inclusion criteria were (1) research that examines organisations, people and actions whose primary intent is to promote, restore and maintain health and health equity via appraisal of at least one of the six WHO health system building blocks; (2) data collected, produced by or about one or more NGOs have been used to investigate the research question; and (3) published in English in a peer-reviewed publication. The use of NGO data was determined by searching the main text and reference lists for references to NGO data and assessing how it had been used in the study/article.

The classification of NGOs can be problematic and there is considerable debate surrounding the taxonomy of NGOs. However, there is broad agreement that NGOs can be defined – and are for this paper – through the following shared structural and organisational features: (1) private or non-state; (2) self-governing; (3) formalised; and (4) not-for-profit organisations [58]. Multilevel (a mix of NGO and state/regional government agencies) and humanitarian organisations, such as the UN, WHO, and International Committee of the Red Cross, are exempt from this definition as their legal status and roles are distinct from that of NGOs. Accordingly, data collected and produced by these organisations were excluded from our review.

### Stage 4: charting the data

Information from the included studies was ‘charted’ by the lead author (SM). This is the term used by Arksey and O’Malley to describe the process of identifying, recording and organising key items of information from each study according to key issues and themes. To enable comparisons between many studies with diverse study designs and contexts, the following information was recorded for each study: NGO role in the publication – author/co-author/other contribution; geographical context – research setting (country/region); developed/developing country; HPSR area – the goal and applicable WHO building blocks; study details –design (e.g. literature review, case study, evaluation); study about NGO activities (e.g. NGO programme evaluation) (Yes/No); primary data collection in addition to secondary analysis (Yes/No); NGO data – which named NGOs are referenced; data type (e.g. an unpublished report, administrative information about the NGO or NGO-collected data such as patient records); data use (e.g. providing context, case study or quantitative analysis); and health category – according to the Health Research Classification System (HRCS) [59].

Following the initial charting exercise, an additional data extraction exercise was performed that focused on one of the data categories – studies with secondary analysis of NGO-collected clinical data. To enable a more in-depth appraisal of the strengths and limitations of the NGO data and its secondary analysis in HPSR, data on the study outcomes, data strengths and limitations were extracted.

### Stage 5: collating, summarising and reporting the results

Given the expected number of included studies with minimal secondary analysis of NGO data, a pragmatic approach to presenting the data was taken. Summaries of different depths are provided to give an overview of the limitations and opportunities of secondary analysis of NGO data in published HPSR and more broadly to highlight the assumption of its primary use (in the form of unpublished NGO reports) as a supporting reference and not as a potential source of data for more in-depth analysis, as follows:

1. a brief descriptive summary of the corpus of included studies – as a scoping review, this serves to show the expected scale of the underuse of NGO data in HPSR by summarising the extent to which studies in this field use NGO data solely or mostly as a contextual reference;
2. a more detailed description of the studies which analysed the NGO data to some extent (excluding studies that only used NGO data in the form of a report as a contextual or corroboratory reference) – this stage seeks to showcase the diverse types of NGO data that have been used for HPSR, where these data originate and how they are being used; and
3. a qualitative analysis of the studies that performed analyses on NGO-collected clinical data – this stage enables a more in-depth investigation of the opportunities for the secondary analysis of a specific type of often rich data collected by health NGOs, when and who is using these data.

During the study selection and data extraction stages (3–5 above), the lead author (SM) made notes reflecting on trends observed in the use of NGO data in HPSR and the difficulties identifying NGO data and their use (e.g. unclear attribution of data to NGOs). We provide a brief summary of the opportunities and limitations of secondary analysis of NGO data that emerged as themes in these notes. A discussion of the implications of the results, gaps and opportunities follows.

## Results

**Fig. 1:**
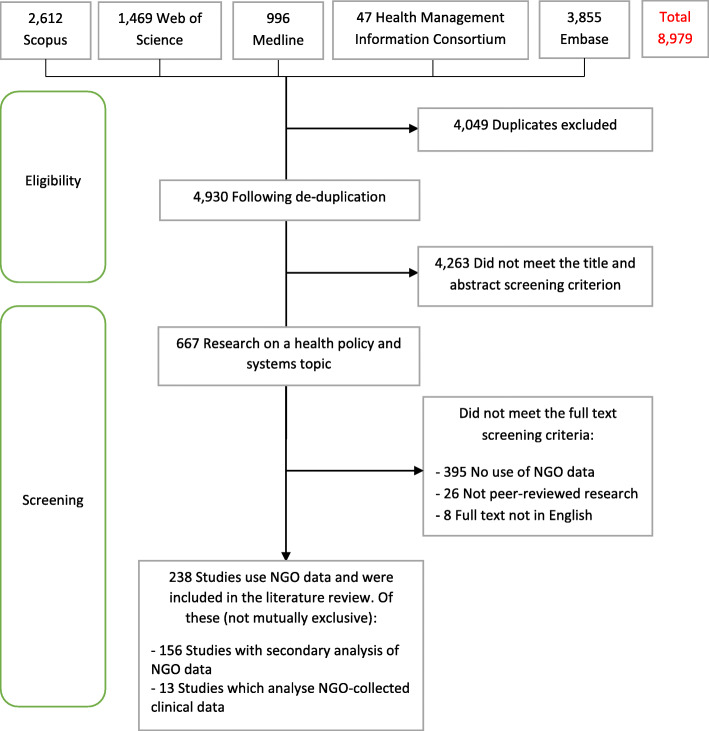
Flow diagram of the process of study selection

**Fig. 2:**
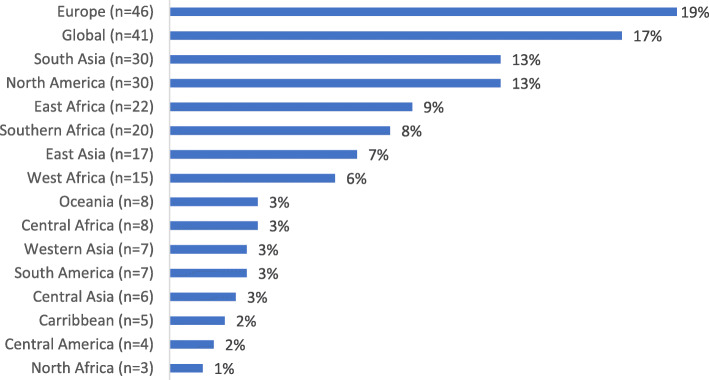
Geographical context of the included studies by region. *n* = 238 studies; regions not mutually exclusive

**Fig. 3:**
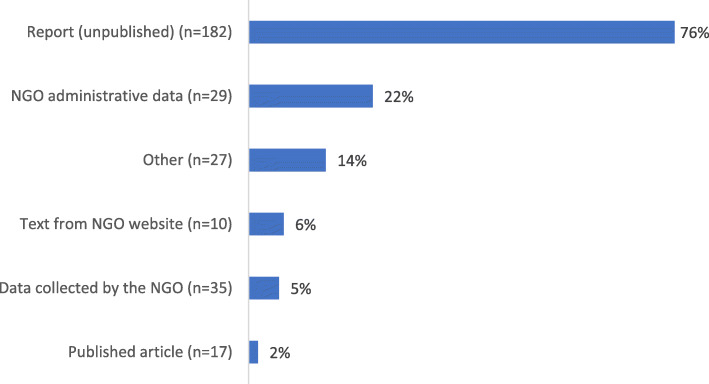
The types of NGO data used in the included studies. *n* = 238 studies; types of data not mutually exclusive. The ‘other’ category includes press releases, clinical guidelines and workshop proceedings

**Fig. 4:**
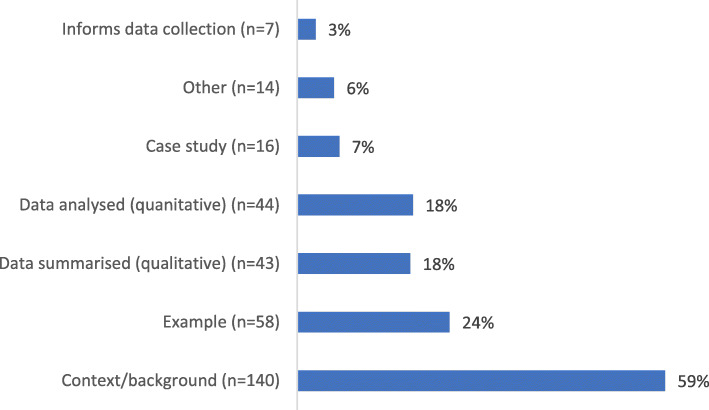
The methods of data use in the included studies. *n* = 238 studies; methods of data use not mutually exclusive. The ‘other’ category includes using NGO data (e.g. reports) as guidance for programme development or to provide a definition

**Fig. 5:**
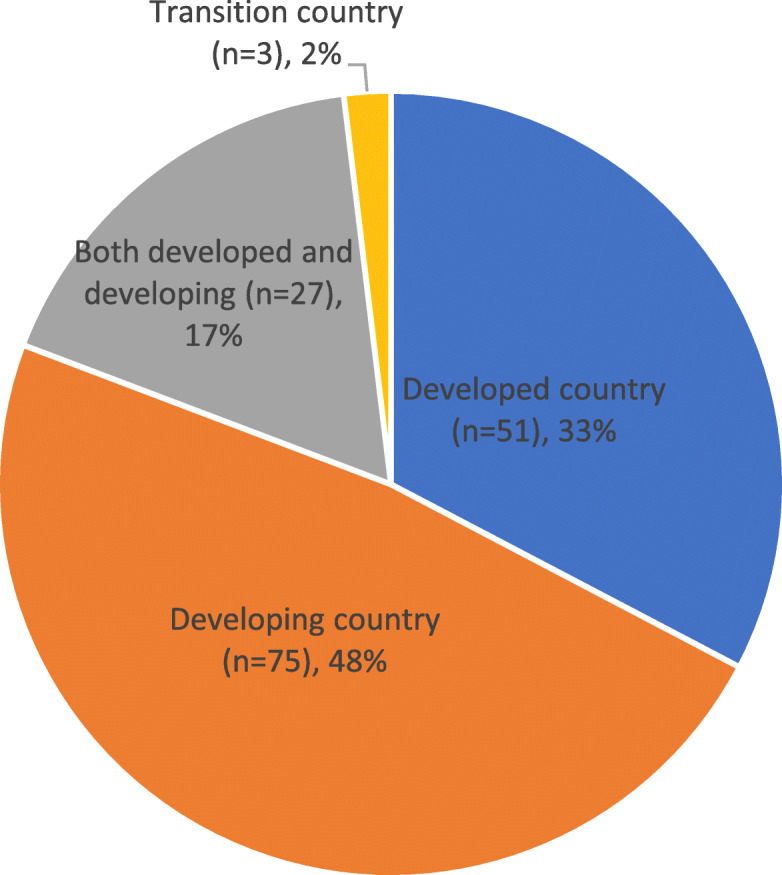
Research setting by UN World Economic Situation and Prospects Categorisation. *n* = 156 studies

**Fig. 6:**
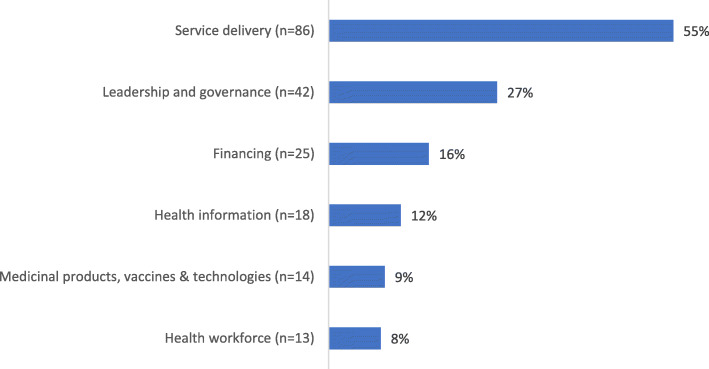
Classification by research area using the WHO health system building blocks framework [25]. *n* = 156 studies; research areas not mutually exclusive

**Fig. 7:**
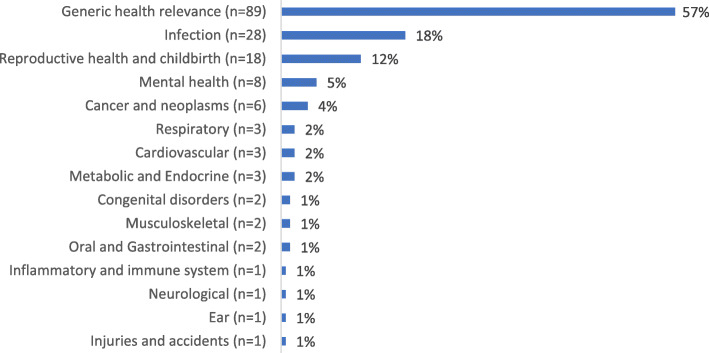
Categorisation of the studies by clinical area using the Health Research Classification System. *n* = 156 studies; clinical areas not mutually exclusive – 10 studies (6%) investigated two or more categories

The search produced 8979 records, of which 238 studies (2.7%) used NGO data to investigate an HPSR topic (Figs. 1, 2, 3 and 4) and were included in the review. Of these, 156 (66%) performed some secondary analysis of NGO data (Figs. 5, 6 and 7); 13 included secondary analyses of NGO-collected clinical data (6% of all studies; 8% of the studies performing secondary analysis) (Table 2).

**Table 2:** Summary of the characteristics and data used in the studies with NGO clinical data (*n*=13)

Author	Country	Name of NGO (author/co-author)	NGO data (^a^additional NGO and/or other data used in the study, including data collection for the purposes of the study)	Study population	Outcomes using the NGO data	Strengths and limitations of the data reported in the article)
Bini et al., Pharmacoepidemiological Data from Drug Dispensing Charities as a Measure of Health Patterns in a Population not Assisted by the Italian National Health Service [60].	Italy	Banco Farmaceutico	Drug dispensation records (includes gender, macro-region of birth, age, duration of the illness)	Low income population not assisted by the Italian National Health Service	Highlighted differences in health between those that do and do not receive Italian NHS assistance	Strengths: large dataset (87,550 subjects); complete data Limitations: individual patient data not provided so analysis by group not possible
Carlson et al., Inequitable Access to Timely Cleft Palate Surgery in Low- and Middle-Income Countries [61].	Ghana, Ethiopia, Democratic Republic of Congo, and Madagascar, China, India, Nicaragua, Bolivia, Paraguay, Peru, Mexico	Operation Smile (co-author)	Patient records (includes gender, age, diagnosis, proposed surgical repair, and documented operation)	People without access to cleft palate/lip in low and middle income countries	Highlighted inequalities in access to surgical care	Strengths: comprehensive initial consultation so could select a sample with specific characteristics Limitations: no high income group comparison data
Cunningham et al., Occupational Therapy to Facilitate Physical Activity and Enhance Quality of Life for Individuals with Complex Neurodisability [62].	UK	Royal Hospital for Neuro-disability (authors)	Patient and therapist records	Individuals with complex neurodisability and limited physical activity	Demonstrates the role of occupational therapists and meaningful physical activity for people with neurodisabilities	Strengths: none reported Limitations: none reported
Deboutte et al., Cost-effectiveness of caesarean sections in a post-conflict environment: a case study of Bunia, Democratic Republic of the Congo [63].	Democratic Republic of Congo	NGO name not reported	Patient records (includes maternal deaths and obstetric care)^a^	People with limited access to obstetric care in a conflict-affected country	Highlighted challenges to service provision during transition from NGO to national health system healthcare, with the need for additional support from NGOs to ensure equitable access	Strengths: adequate data to compare the obstetric characteristics of women who lived in the same neighbourhood and delivered around the same time (e.g. caesarean section versus virginal delivery) Limitations: limited generalisability of the findings to other crisis situations e.g. sudden-onset natural disasters
Gurung et al., Large-scale STI services in Avahan improve utilization and treatment seeking behaviour amongst high-risk groups in India: an analysis of clinical records from six states [64].	India	Avahan (delivered by a network of NGOs) (co-authors)	Individual clinical monitoring data (includes sex, age, years in sex work, symptoms, diagnosis)^a^	High risk groups for sexually transmitted infection	Demonstrated the need for services by high risk groups and the ability to provide treatment at a large scale	Strengths: none reported Limitations: incomplete data (missing dates, site, ID number)
Jacobs et al., From public to private and back again: sustaining a high service-delivery level during transition of management authority: a Cambodia case study [65].	Cambodia	Enfants et Développement project taken over by Swiss Red Cross (SRC) (CRC co-author)	Patient data (includes child vaccination and birth-related information)^a^	People without access to health services during transition to a national health system	Demonstrated how transition from NGO to public service delivery can be monitored and achieved without a loss in service capacity and quality	Strengths: none reported Limitations: lack of controls for comparison with the study sample
Kohli et al., A Congolese community-based health program for survivors of sexual violence [66].	Democratic Republic of Congo	Foundation RamaLevina (FORAL) (co-author)	Patient records (includes demographics, experience of sexual violence, physical and mental health problems, treatment)^a^	Survivors of sexual violence in a conflict-affected country	Demonstrated the need and ability of mobile health services to support and strengthen existing services by reaching rural and conflict-affected populations	Strengths: none reported Limitations: limited data collected as new clinical form designed to minimise the burden of documentation for patients and clinicians
Lindgren et al., Using mobile clinics to deliver HIV testing and other basic health services in rural Malawi [67].	Malawi	Global AIDS Interfaith Alliance (GAIA) (co-author)	Patient data (presenting illness)^a^	Rural communities without access to HIV services	Demonstrated the need and effective monitoring of mobile clinics in remote rural villages and seasonal variation	Strengths: clinical forms well-matched with the government-run health centre records so comparison possible Limitations: inconsistent data recording (e.g. not all sites distinguished between dysentery and diarrhoea)
Marsden et al., Risk adjustment of heroin treatment outcomes for comparative performance assessment in England [68].	UK	NGO name not reported (NGO-run services contribute data to the national monitoring system)	Drug treatment records (includes history and current substance use, health and social functioning, demographic information)^a^	Substance users in a high income country	Highlighted variation in good and poor practice across the UK so inequalities can be addressed	Strengths: comprehensive individualised data which can be stratified by site Limitations: none reported
Odwe et al., Introduction of Subcutaneous Depot Medroxyprogesterone Acetate (DMPA-SC) Injectable Contraception at Facility and Community Levels: Pilot Results From 4 Districts of Uganda [69].	Uganda	Reproductive Health Uganda	Patient records^a^	Women receiving contraceptive services	Quantified the volume of contraceptive methods provided at NGO clinics	Strengths: none reported Limitations: absence of unique patient identifiers for data from every clinic (including village health teams and mobile outreach).
Poenaru, Getting the job done: analysis of the impact and effectiveness of the SmileTrain program in alleviating the global burden of cleft disease [70].	Global	SmileTrain	Patient records (includes surgical procedures)	People without access to cleft palate/lip in low and middle income countries	Highlighted the global burden of disease caused by delayed surgery	Strengths: large multi-country dataset Limitations: dataset needs to be combined with additional data sources for verification; not representative of the LMIC cleft palate/lip population as 79/171 LMICs represented
Ruckstuhl et al., Malaria case management by community health workers in the Central African Republic from 2009–2014: overcoming challenges of access and instability due to conflict [71].	Central African Republic	The MENTOR (co-author)	Community health worker records (includes basic demographic information, symptoms, test results, treatment)	Malaria-endemic region of a conflict-affected country	Highlighted specific local context issues: variation in malaria trends between the seasons and during periods of conflict	Strengths: longitudinal data (2009 to 2014) Limitations: Incomplete data (not reported during peaks in violence); data collection tools not implemented across sites simultaneously
Wendland et al., Undocumented migrant women in Denmark have inadequate access to pregnancy screening and have a higher prevalence Hepatitis B virus infection compared to documented migrants in Denmark: a prevalence study [72].	Denmark	Unnamed NGO (which runs clinics providing healthcare to undocumented migrants)	Patient records^a^	Undocumented migrant women aged 18-45	Prevalence of pregnancy and sexually transmitted infection	Strengths: the ability to conduct research in a population who do not engage with national health services Limitations: limited generalisability (do not know if the sample (women presenting to a clinic) were representative of the study population (e.g. more/less healthy)); some missing data (test results)

### Overview of all included studies

This overview describes all the studies that included NGO data in research on a health policy and systems topic (*n* = 238). Most of the studies were conducted in single country contexts (70%; *n* = 166). The remainder investigated HPSR topics across multiple countries (either within the same or different geographical regions, e.g. West Africa (14%; *n* = 34), or from a global perspective (16%; *n* = 37)) (Fig. 2). Unpublished reports produced by NGOs were the most common source of NGO data and were used in 76% of the studies (*n* = 182; Fig. 3).

The NGO administrative data analysed in 22% (*n* = 29) of the studies consisted of databases of NGOs, service information (what service was provided, to whom and where), service evaluation (programme coverage rates, outcome indicator prevalence), and NGO financial accounts (e.g. [73]). Other types of data analysed (14% (*n* = 27) of the studies) included project plans, operational guidelines and data, e.g. a framework for health needs assessment (*n* = 7) [74–80], press releases and news stories (*n* = 5) [81–85], definitions produced by NGOs (used as operational constructs in the studies) (*n* = 4) [86–90], clinical guidelines and guidance on clinical guideline development (*n* = 4) [74, 80, 91, 92], educational resources and information for clinicians (*n* = 3) [93–95], unspecified contributions of background information by NGO members (*n* = 3) [96–98], contracts (*n* = 3) [76, 77, 99], an interview transcript (*n* = 1) [82], and workshop proceedings (*n* = 1) [100]. The clinical data analysed in 5% of the studies were collected by NGOs via drug distribution and treatment monitoring systems and health monitoring information systems (patient records).

Half the studies (50%; *n* = 120) exclusively performed secondary analyses of available NGO data. The remainder performed additional data collection exercises for the purposes of the study (*n* = 118). Many of the studies were either literature reviews or had an initial review component (34%; *n* = 81). As in the wider set of studies, there was variation in the use of NGO data in these review elements. Only a minority (35%; *n* = 28/81) found and included grey literature (i.e. unpublished reports) in the analysis (e.g. [26, 88, 101]) – the majority excluded unpublished NGO-produced reports. Others included means of identifying relevant grey literature in their search strategies but did not find or exclude NGO reports at the screening stage [102–104].

More commonly, NGO-produced reports were used only as a supporting (corroboratory) reference or to provide a contextual detail, such as a statistic about the study population. For example, in a document analysis investigating armed conflict in Pakistan and the role of NGOs in restoring health services, a report by (the NGO) International Crises Group was used to support the statement, “*the destruction of health centers and killing and kidnaping of doctors by the terrorists have made it more complicated for the locals to access basic health facilities*” [105]. Neither this, nor any other NGO reports were included in the analysis. This is an example of corroboratory reference use. In a study on the contributions of aid organisations and international NGOs to health in Nepal, an NGO report was used to state that Nepal is ranked 146th out of 178 countries on the Corruption Perception Index [106]. No NGO resources were included in the literature review component of the study. This is an example of contextual reference use.

Of the studies, 35% (*n* = 82) had more than one different use of NGO data (e.g. as a supporting reference in the introduction and NGO-collected patient data in the main analysis) (Fig. 4). As well as a range of different types of NGO data used (qualitative and quantitative), the extent of the secondary analysis of NGO data varied from cursory to in-depth.

### Overview of included studies with secondary analysis of NGO data

The following summary is only for those studies that performed secondary analysis of NGO data, excluding studies that only used NGO data as a corroboratory or contextual reference (*n* = 82 omitted (36% of the 238 studies included in the review); *n* = 156 included). Almost half of the studies (*n* = 156) investigated HPSR topics in developing countries (Fig. 5) [107].

Using the WHO health system building blocks framework, most of the studies (55%; *n* = 86/156) had the goal of improving health via research on service delivery (Fig. 6) [4]. For example, via evaluation of the role of the NGO in health system delivery or the efficacy of scaling-up an NGO-delivered service from the regional to national level (e.g. [76, 108, 109]). The majority focused on one building block (81%; *n* = 127/156); four examined all six [26, 70, 110, 111].

There were studies relating to 15 of the 21 HRCS categories. Most of the studies covered topics of generic health relevance (57%; *n* = 89), followed by infection (e.g. HIV, tuberculosis, sexually transmitted infection; 18%, *n* = 28), and reproductive health and childbirth (10%; *n* = 16) (Fig. 7). Eight were on mental health topics (only two were conducted in developing countries – West Africa and Lebanon [89, 112]).

NGOs had no stated involvement in the publication or funding of 67% of the studies (*n* = 105). NGOs were the sole author of 6% (*n* = 9) or co-author of 20% (*n* = 31) of the studies and funded either the study or the researcher in 7% (*n* = 11). Most of the studies with NGO authors or co-authors included secondary analysis of at least one source of their own data (78%; *n* = 31/40). NGO funding of the studies authored by NGOs can be assumed and is likely (but was not reported) for some of those co-authored.

### Summary of the findings for studies that used NGO-collected clinical data

For a more detailed investigation of the use of NGO data in HPSR, this summary presents the findings of the studies that performed secondary analysis of clinical data collected by NGOs (*n* = 13).

NGO clinical data are data collected by NGOs about the health of people using their services (e.g. patient records) and not about their own activities (e.g. NGO accounts and performance monitoring systems). These data, sometimes collected over a period of many years and often in populations without access to a national health system, result in unique and longitudinal datasets, which can be used in a range of exploratory and comparative studies [65, 113], for example, to examine how the health and healthcare use of marginalised population groups is different from national patterns, how they change (in health, budgets and service provision) over time and seasonal variations [23, 60, 114–117].

Of the 13 studies in this review that performed secondary analysis of NGO-collected clinical data, 69% (*n* = 9) were studies in developing countries. As in the preceding overview of the studies performing secondary analysis, most of the studies using clinical data investigated service delivery-related research questions (69%; *n* = 9). Of the HRCS categories, most were of generic relevance (39%; *n* = 5), followed by infection (23%; *n* = 3) and reproductive health and childbirth (15%; *n* = 2). The characteristics and use of NGO data in these studies are summarised in Table 2.

### Emergent themes

Notes reflecting on the studies and NGO data identified were made by the lead author during the screening process and analysis of the 238 studies and were discussed with the co-authors. To conclude the results section, emergent themes from these notes and discussions are described with indicative references. The themes largely arise from the disjunct between our understanding of the significant role of NGOs in health policy and systems and the perceived underuse of these data, given the sheer amount being collected and generated by health NGOs but not used in research.

Indeed, several studies included in our review were either aimed at ascertaining just how great the contribution of NGOs was in specific fields, (e.g. surgical practice, cancer care and lesbian gay bisexual and transgender health services [118–120]) or the scope for greater collaboration between NGOs and others (e.g. business, multilateral and other NGOs) [45, 81, 121, 122]. This research derives from the knowledge that there are large numbers of health NGOs worldwide but there is limited knowledge of the extent of their activities (outside the organisation) [105, 123–125] and barriers to partnerships [81, 122, 126].

When grey literature was included in reviews or as background information, we observed a tendency for authors to look to large, usually international and sometimes national, NGOs and multilevel organisations for information in the form of reports, rather than looking for information from small but potentially highly relevant regional (or national) organisations (e.g. [88, 127–129]). None of the studies that report searching NGO websites for relevant documents list the NGOs or search strategy used (e.g. [102, 130]). Furthermore, we noted that even studies wholly or partially about NGOs and their health-related activities sometimes did not include (or attribute) any NGO-produced or collected data (e.g. [131–135]). For example, one study exclusively reviewed grey literature on the mental health and psychosocial response to the 2015 earthquake in Nepal, which they obtained through online information-sharing platforms and response coordinators. Although this paper mentions the work of NGOs, and we can assume that some of the 168 documents included in the review were produced by NGOs, there is no attribution of these resources (therefore, this review was excluded from our study) [136]. Developments in these areas could both provide opportunities to improve health in the communities where NGOs operate and facilitate HPSR via data sharing and influencing data collection.

#### NGO data use

NGOs that are embedded in a community or act in the context of an emergency or crisis and provide a valued service, are likely to be trusted and have access to key stakeholders, enabling exploratory research on sensitive or contentious issues [66, 117, 137, 138]. The collection of data ‘in the field’ enables evaluation of the efficacy of interventions and services in the real world and differing clinical settings, adding to the data from clinical trials and to support service scale-up [61, 139–141]. These data can also be used to show the extent to which health systems and other development targets are being met (e.g. by mapping changes in health outcomes against development goals), by highlighting deficits and increasing pressure for these goals to be achieved [26, 142, 143]. However, only two papers used NGO data for performance monitoring in this way [26, 144] and none referenced Sustainable Development Goal (SDG) 3, which outlines targets to ensure healthy lives and promote wellbeing at all ages [145].

Although there were examples of the secondary analysis of NGO data, the number of studies doing so was relatively small given the amount of data inevitably collected and/or generated by NGOs. The effective use of NGO-collected and produced data in published HPSR shows that NGO data can be accessed and used by researchers to answer HPSR questions but is largely not [146]. NGOs with a research agenda, who might be more aware than academic institutions of the data collected by other NGOs or have established partnerships with other NGOs (e.g. joint service delivery or members of the same NGO network organisations), also appear reticent to use other NGOs’ data. For example, of the studies with NGO authors or co-authors (indicating a research agenda/interest), only four (15.4%; *n* = 26) either performed a secondary analysis of data collected by another NGO or referenced literature by other NGOs [86, 108, 147, 148]. One of the included studies found that NGOs may be less likely than the academic and public sector to draw on the expertise of others, including other NGOs, in the production of Health Impact Assessments [149]. The same could be true for other areas of health research.

#### Identification and limitations of NGO data

It was not always easy to identify which NGO’s data were being used and where the data had been acquired. In the studies, it was common practice to name an NGO and state their aims, scope, etc. but not to link to their websites, thereby not attributing the (most likely) source of the data (e.g. [132]). It was often not clear how much of the data were provided by NGO-delivered services, particularly when documents or case studies were analysed (e.g. [68, 96, 150, 151]). These practices result in the under-acknowledgment of NGO data in HPSR studies. An example of appropriate, yet limited, attribution was demonstrated by Cancedda et al. [110], who referenced the ‘Our Work’ section of the website for the NGO used as a case study (and co-author) in the research article. However, it remained unclear whether the co-author from the NGO (Partners in Health) was the primary source of the data used in the evaluation or whether NGO-produced (internal and/or external) documents and the website were the main source.

Limitations of NGO data were reported in some of the 238 included studies. In some instances, NGOs may be commissioned or tendered to provide a service within a country’s national health system. The synthesis of data across NGO and health system services was hampered by using different record-keeping systems that did not all record the same data or in the same way or data recording was incomplete for some services [77, 115, 152, 153]. This issue may be even greater where services are rolled out to new communities without consistent record-keeping and electronic data monitoring systems [64, 115].

Data collected by NGOs in challenging geographical areas or amid humanitarian crises were largely designed for practice and not for research [63, 67, 147]. There may be limited data collection and incomplete or inaccurate data [65, 72, 116], or researchers may not be fully aware what data have been collected and may be available. Elements of rigorous research are often neglected as an NGO’s first consideration is to treat and support the population in need by acquiring only essential information [38]. Adequate descriptions of the context, intervention and/or strategy, control groups and randomisation for intervention studies and generalisability were largely absent in the studies [71, 116, 154].

However, we note that claims about data limitations attributable to NGOs are not valid in all contexts. For example, health surveillance information collected by NGOs and other healthcare providers in the Central African Republic is inconsistent as disease screening programmes have been erratic in some regions due to security issues [155]. In a study on undocumented migrants visiting health clinics in Denmark, the generalisability of the findings was limited by the lack of data for the wider undocumented migrant population [72]. In both instances, the data limitations were outside the control of the NGO that provided the data for the studies.

Knowledge of these data limitations or concerns about the quality of the research using them are possible explanations for the limited use of NGO data in HPSR.

## Discussion

This review is the first, to our knowledge, to use a systematic method to provide a comprehensive examination of how data collected and produced by NGOs are being used in HPSR and the extent to which secondary analyses of these data are being performed. We found evidence of the analysis of NGO data in HPSR in 66% of the studies included in the review (*n* = 156). NGO-produced reports were the most common form of data used (in 64% of the studies) but with limited analysis of these data (e.g. their use to critique the NGO’s activities or provide a brief example). Only 8% of the studies performed detailed analyses that used clinical data collected by NGOs. When the scale and diversity of NGO practice (and therefore data collection) and the potential value of NGO data to research are considered, our results indicate limited use of secondary analysis of NGO data. The use is limited both in the quantity of studies and the depth of analysis.

For a majority of HPSR topics, relevant data are being collected by NGOs and could be used to answer, or contribute to answering, research questions of relevance to both NGOs and academics either as a primary or supplementary data source (i.e. action research). The opportunities for NGO data use lie far beyond the use of unpublished reports as supporting references. Our study highlights the frequency of this minimal use of NGO data as well as some innovative uses of NGO-produced data, for example, NGO administrative data to assess the scale (and spending) of NGO operations to learn more about the contribution of NGOs to world health [77, 108, 156, 157]. We highlight the value of NGO-collected data for research in hard-to-reach populations, including undocumented migrants, people experiencing domestic violence and in conflict-affected areas (e.g. [105, 117, 158, 159]). Therefore, while some researchers are accessing and performing secondary analysis on NGO data, it is our view that real and perceived barriers to NGO–academic collaboration and NGO data access result in the entrenched underuse of NGO data in HPSR. This view is shared by others working in development research [7, 11, 15–17, 32, 48, 50, 57, 160].

As expected, NGO data use in HPSR, beyond inclusion as a corroboratory or contextual reference (*n* = 156 studies), occurred in studies with NGO authors/co-authors (26%), in developing countries (48% of studies), with the aim of evaluating a service delivered by NGOs (55% of studies), and in clinical areas of generic health relevance (57%). Our review was not able to identify why researchers do not perform secondary analysis of NGO data, although some of the challenges that they face when using NGO data were highlighted in Table 2, namely incomplete, inconsistent or aggregated data and lack of control/comparison groups drawn from the same population. We can, however, identify opportunities based on examples of the successful use of NGO data and suggest how its underuse might be mitigated to encourage more routine use of this largely untapped but highly valuable resource. The following discussion draws on the studies included in our review and the wider literature on NGO data and NGO–academic collaboration.

### Opportunities for NGO data use

The population groups for which secondary analysis of NGO-collected clinical data (*n* = 13) were performed were all marginalised groups with restricted access to healthcare services, such as rural communities, people affected by conflict, and violence or drug addiction. It is highly likely that the clinical data collected by NGOs, whilst providing a valued healthcare service, were not collected for research purposes but were the best, and possibly only, source of data for these groups [6, 13]. Therefore, using NGO data (and collaboration with NGOs to collect data) is an opportunity to raise awareness of health issues in groups who are often overlooked or hard to reach by academic researchers [38, 161]. This may be the case especially in developing countries with challenging political and/or cultural contexts and where there may be stigma associated with certain health conditions such as sexually transmitted diseases [11, 66, 117]. NGOs may also be in a better position, in terms of trust, to obtain these sorts of data [113].

Greater use of NGO data could have a particularly important role to play in increasing awareness of health conditions, health needs and health service use for marginalised groups and reducing the inequalities experienced by these groups. For example, the use of NGO data could provide new insights into disparities in the health of marginalised groups compared with the general population, which could inform the development of policy and potential interventions, as well as being used more extensively in programme and facility evaluation and advocacy [38, 109, 162].

Some of the studies we analysed used longitudinal NGO data. These are extremely valuable for monitoring changes in health over time and are important in the context of determinants of health, including changing social, economic and environmental conditions [163]. Longitudinal data are especially valuable when environmental and political changes are occurring at an unprecedented rate such as in humanitarian crises. Conducting long-term studies has substantial cost implications that all organisations face. Accessing available longitudinal data sets produced by NGOs can facilitate the research of interest whilst limiting the costs for research institutions [6]. In other cases, NGOs work with a community for a relatively short period of time, ceasing activity when donor funding ends. It is important that the benefits of these data are realised, not least because demonstration of impact can support requests for further funding [54, 160, 164].

Longitudinal data are also important for conditions that develop over time or may be rooted in childhood or mental health conditions [141, 163]. However, only eight of the studies that performed secondary analysis on NGO data (*n* = 156) addressed mental health topics and only two in developing countries. This is perhaps not surprising as, despite mental health accounting for 27% of all years lived with disability worldwide, mental health has received far less interest in research and practice in developing than developed countries [165, 166]. The evidence of an absence of available (including NGO) data in these contexts can also help build the case for funding for, for example, the scaling up of NGO service delivery and research activities (including to collect better quality data) in these (developed and developing country) locations [45, 167, 168]. The assumption (and sometimes reality) of poor-quality data is a common academic explanation for not attempting secondary analysis of NGO data [8]. However, it is not true of all NGO data [148]; for example, the NGO, Reproductive Health Uganda, provides training on data collection, storage and reporting to ensure minimum data standards across their network of 17 health clinics [69]. Entering into collaborations with NGOs working in the field of interest can benefit both partners in their shared aim of improving health outcomes [11, 17, 167]. NGO data and NGO–academic collaboration can be particularly valuable in action research, especially within the contexts of refining approaches to achieve the SDGs and developing research methods to collect high quality data in challenging settings [48, 49, 52]. HPSR is increasingly using the SDGs as a framework for agenda-setting [8]. NGO data can be used for measuring progress against SDG targets, not least because health intersects with many other areas of development [36, 145, 169].

Collaboration could also help the HPSR based on NGO data to be disseminated faster, especially in disaster or conflict areas. For example, most of the operational research on the Ebola outbreak from March 2014–December 2017 was published after the WHO had initially declared the outbreak over in January 2016 rather than during the outbreak [170]. NGO–academic collaboration could possibly have enabled analysis and dissemination of the data from these contexts to the Ebola research community and NGOs operating in the field sooner, more rapidly advancing understanding of the disease and policies to respond to outbreaks [170].

The benefits of collaboration for academics include accessing NGO data that provides them with an opportunity to influence data collection tools and methods to improve data quality and relevance for their purposes [11]. For example, academics can work with NGOs to help ensure that data are collected in a way that means they are consistent over time and can be used for temporal analysis [63, 67]. Academics need to recognise the potential of the data whilst accepting the inevitable limitations of data collection by non-academics in challenging settings, with changing social and environmental landscapes, aiming to collect only essential information [64, 66, 71, 113].

The lack of experience of many NGOs in data handling and management can also limit the further use of their data, but this is another area where there can be positive sharing of best practice leading to improved capacity over time [13, 15, 32]. In addition, for researchers, secondary analysis provides the benefit of being able to assess data quality prior to performing the analysis [148]. The process of appraising NGO data has time and cost implications, but much can be learnt by academics, such as what additional data collection activities are required and how their research questions can be adapted based on the available data [34].

Through collaboration, an NGO develops its research capacity, the ability to evaluate its activities and can influence its partners’ research agenda. The ability to produce evidence of local health needs and deficits in service provision can also strengthen NGOs’ advocacy for health policy and funding reforms from governments and donors [6, 57]. Developing research (and importantly, evaluation) capacity has been shown to facilitate the sustainability and scaling-up of NGO activities [167, 171, 172]. However, in the year 2000, only 23% of 37,000 international NGOs were performing research activities (no more up-to-date data were available). Explanations for limited or no collaboration with academic institutions include suspicion of the academics’ agendas (including competing interest and power inequalities in decision-making about the ethics, purpose, application and dissemination of the research [7, 32, 173]); power and global north–south inequalities [174–176]; and doubts about the value of communicating with academic audiences [167, 171, 172]. Issues of competing interests can arise between what data collection are considered necessary by the NGO (e.g. to treat or monitor health in the population) versus by the academics (i.e. to produce high quality, publishable research), the ownership of this data and linkage between new and existing data collected [32, 173, 177, 178].

We advocate for greater NGO–academic collaboration. The sharing of data could work both ways as, through collaboration, NGOs that perform research could benefit by accessing other datasets such as those collected by academic or public sector institutions [179]. NGO–NGO, NGO–business and NGO–public sector collaborations can also help develop research capacity (and programme evaluation), thus facilitating the collection and use of NGO data in HPSR [121, 172, 180, 181].

Developing and implementing data standards and protocols to be adopted by (or together with) NGOs could be a way to enhance the wider use of the data they collect. Secondary data analysis requires the NGOs to grant permission and re-issue data for another purpose than originally intended. We are aware of the increasingly stringent ethical requirements constraining research organisations [182, 183]. The absence of ethical standards informing the collection and management of data by NGOs (or equivalence of this governance to the standards used by research institutions) may prohibit the use of NGO data by academics [184]. The need for standardised data protection and for inter- and extra-NGO sharing procedures is a current debate for humanitarian and development agencies [185, 186]. Given the potential importance of NGO data for raising attention of marginalised groups and calls for data interoperability (joined up data) to achieve development goals [187], differences in methodological protocols and data standards can prevent the conditions of marginalised groups being brought to wider attention [50]; this could maintain inequalities or even exacerbate them.

During the review, we found many instances of the under- or ambiguous acknowledgement of NGO data in various forms. Some studies stated that the data were provided by an NGO or that a number of NGOs had been involved in the data collection but did not provide their names. Examples include referring to “*the NGO forum of Cambodia*” (comprised of several unnamed NGOs) [188], stating that “*six of the nine data providers in the study were NGOs*” but giving no further details [77], and acknowledging contributions by NGOs but not stating whether they provided data [97]. Elsewhere, NGO activities were used in case studies or given as exemplars (e.g. [96, 189]), sometimes using information from their websites (e.g. [190]). Oftentimes, these data were either not attributed in the references (thus the study becomes the de facto data source) or the reference was for another source where the data had been reported (i.e. not the original source of the data) [189]. Two studies refer to data on funding received by NGOs but, as they do not reference the source(s) of this NGO administrative data, the study becomes the source [106, 126]. Further, when an example of a specific NGO activity is used in a WHO publication and this publication is referenced, WHO becomes the data source and not the NGO.

By not attributing the NGO directly, inequalities of knowledge and power between NGOs and research organisations, multilevel or network organisations such as WHO, the UN, World Bank and are upheld [53]. This failure to attribute research to NGOs was also seen when the Global Health Watch Report 4 [191], which has NGO and NGO network co-authors (e.g. Health Action International and the People’s Health Movement), was cited [23]. However, it is worth noting that collaboration between NGOs and multilevel organisations does enable NGO research capacity and can raise the profile of their activities. For example, Kilic et al. [192] refer to documents on the healthcare system monitoring activities of the Turkish Diabetes Foundation, which were jointly published by the Turkish Ministry of Health, WHO Europe and the International Diabetes Federation of Europe. The multinational and multilevel organisations may have assisted the Turkish Diabetes Foundation with publication if they did not have the capacity or resources to publish these documents independently. Their support with dissemination could also greatly increase the reach of these documents. Whatever the reason for this and similar NGO–multilevel organisation collaborations, we would stress the need for greater transparency in data use, clarity in relation to source attribution, and appropriate and full acknowledgment of NGO data and contributions [32].

Our analysis has focused on published research literature, but NGO research is more prevalent in grey literature, as demonstrated by the number of studies using unpublished NGO literature as contextual or corroboratory references. The relevant grey literature is harder to locate, leading researchers to use the pragmatic strategy of reference list searching and looking for relevant documents on the websites of organisations that they know to work in the field of interest [79, 193–196]. This practice perpetuates imbalances in the visibility of research by large NGOs and multilevel organisations compared with smaller, less well-known NGOs [197]. There are search tools and guidelines for searching grey literature, but the academic preference for using peer-reviewed published literature in formal reviews remains [198]. If grey literature was more routinely included in literature reviews in HPSR, researchers would find more NGO-produced (unpublished) reports that may contain valuable data for inclusion in literature syntheses. The inclusion of these references could be particularly beneficial in areas with little published research (e.g. in marginalised groups) or areas dominated by published research from high-income countries (e.g. mental health). Given the more common use of reports produced by multilevel or international NGOs, systematic searches of grey literature and use of the unpublished data identified could also help raise awareness of research activities by smaller NGOs. This increased exposure could also help them attract funding and academic collaborators to grow their research capacity [15].

We recognise that a review of this nature inevitably has limitations. We performed a literature scoping exercise across a range of interdisciplinary and health-specific databases, favouring a broad search strategy in a few key databases rather than a more focused strategy in every potentially relevant database. We may also have missed some relevant studies due to the under-acknowledgement of NGO contributions, the challenge of identifying whether named organisations fitted the NGO definition and the inclusion of only studies published in English. All of this points to the importance of further research in this field to examine in more depth the value of different types of NGO data identified here but not investigated in detail. Greater rigour in data sharing agreements and more systematic access to the data collected by NGOs is also important. Additionally, the comparison of practices of NGO data use in other areas of development research could help researchers begin to mitigate the issues of NGO data use in HPSR, e.g. by adopting best practices and NGO–academic collaboration standards used elsewhere [48].

We gave a broad overview of how NGO-collected and produced data have been used and the extent of the secondary analysis of NGO data in HPSR, with a more in-depth look at the use of NGO-collected clinical data. It was not possible to provide a comprehensive analysis of how each different type of data identified were used, although we identified some examples of innovative uses of NGO-produced data such as NGO accounts. The public availability of electronic data produced by and about NGOs (including news stories, financial information and court proceedings) is a potentially rich seam for secondary analysis by researchers willing to use less traditional data sources.

## Conclusions

In this review, we have given an overview and specific examples of how, in HPSR, NGO-collected and produced data are being used and in what contexts secondary analysis of NGO data is being performed. There were frequent examples of the use of secondary analysis of NGO data in service delivery evaluations, especially in developing countries and when the NGO is the author or co-author of the study. To a lesser extent, we found examples of the use of NGO-collected clinical data and NGO administrative and other types of data published by researchers without any (known) connections to NGOs.

We have argued that given the scale of health NGO operations worldwide, NGO data constitute a vast and valuable source of data for HPSR. Yet, the value of these data is under-realised, and the data underused and under-acknowledged in HPSR. By drawing on the studies included in the review and wider literatures on NGO data and NGO–academic collaboration, we have offered suggestions for routes to the greater use of secondary analysis of NGO data in HPSR. These include the routine inclusion of grey literature in literature reviews and greater NGO-academic collaboration that is informed by clear and agreed standards for research protocols, ethics and data management. With its broad scope, this review offers an entry point for further discussion of how secondary analysis of NGO data can be used more extensively in HPSR and other areas of research driven by development goals.

## Supplementary information

**Additional file 1.** Full list of references for the studies included in the review.

## Data Availability

All data generated or analysed during this study are included in this published article and its supplementary information files.

## Abbreviations

NGO: Non-governmental organisations
HMIC: Health Management Information Consortium
HPSR: Health policy and systems research
HRCS: Health Research Classification System
SDGs: Sustainable Development Goals
