# Supplementary material for: Repurposing NGO data for better research outcomes: a scoping review of the use and secondary analysis of NGO data in health policy and systems research
Source: Health Res Policy Syst. 2020 Jun 8;18:63. doi: 10.1186/s12961-020-00577-x (PMC7278191; doi:10.1186/s12961-020-00577-x)
Supplement: Supplementary file 1 — Additional file 1. Full list of references for the studies included in the review. [file 12961_2020_577_MOESM1_ESM.docx]

# Full list of references for the studies included in the review

## Articles which analysed NGO data (n=156)

1. Abdulmalik J, Fadahunsi W, Kola L, Nwefoh, E, Minas H, Eaton J, Gureje O. The Mental Health Leadership and Advocacy Program (mhLAP): a pioneering response to the neglect of mental health in Anglophone West Africa. Int J Ment Health Syst. 82014. p. 5.
2. Alam, K, Ahmed, S. Cost recovery of NGO primary health care facilities: a case study in Bangladesh. Cost Effectiveness and Resource Allocation. 2010;8:12.
3. Alonge O, Gupta S, Engineer C, Salehi AS, Peters DH. Assessing the pro-poor effect of different contracting schemes for health services on health facilities in rural Afghanistan. Health Policy Plan. 2015;30(10):1229-42.
4. Amador S, Goodman C, Robinson L, Sampson EL. UK end-of-life care services in dementia, initiatives and sustainability: results of a national online survey. BMJ Support Palliat Care. 2018;8(4):424-7.
5. Ancker S, Rechel B. HIV/AIDS policy-making in Kyrgyzstan: a stakeholder analysis. Health Policy Plan. 2015;30(1):8-18.
6. Anggriani Y, Ibrahim M, Suryawati S, Shafie A. The impact of Indonesian generic medicine pricing policy on medicine prices. Generic Medicines. 2014;10(3-4):219-29.
7. Aras, B. Medical Humanitarianism of Turkey’s NGOS: A “Turkish Way?” Alternatives. 2017; 42(4):183-194.
8. Ashford RD, Curtis B, Brown AM. Peer-delivered harm reduction and recovery support services: initial evaluation from a hybrid recovery community drop-in center and syringe exchange program. Harm Reduct J. 2018;15(1):52.
9. Bahuguna P, Mukhopadhyay I, Chauhan AS, Rana SK, Selvaraj S, Prinja S. Sub-national health accounts: Experience from Punjab State in India. PLoS One. 2018;13(12).
10. Baum F, Freeman T, Sanders D, Labonte R, Lawless A, Javanparast S. Comprehensive primary health care under neo-liberalism in Australia. Soc Sci Med. 2016;168:43-52.
11. Bemelmans M, Baert, S, Negussie, E, Bygrave, H, Biot, M, Jamet, C, Ellman, T, Banda, A, van den Akker, T, Ford, N. Sustaining the future of HIV counselling to reach 90-90-90: a regional country analysis. Journal of the International AIDS Society. 2016;19(1):20751.
12. Bennett S, Corluka A, Doherty J, Tangcharoensathien V, Patcharanarumol W, Jesani A, et al. Influencing policy change: the experience of health think tanks in low- and middle-income countries. Health Policy Plan. 2012;27(3):194-203.
13. Bennett, S, Corluka, A, Doherty, J, Tangcharoensathien, V, Patcharanarumol, W, Jesani, A, Kyabaggu, J, Namaganda, G, Hussain, AMZ, Aikins, AD. Influencing policy change: the experience of health think tanks in low- and middle-income countries. Health Policy and Planning. 2012;27(3):194-203.
14. Berman J, Mitambo C, Matanje-Mwagomba B, Khan S, Kachimanga C, Wroe E, Mwape L, van Oosterhout JJ, Chindebvu G, van Schoor V, Puchalski Ritchie LM, Panisset U, Kathyola D. Building a knowledge translation platform in Malawi to support evidence-informed health policy. Health Res Policy Syst. 132015.
15. Berti PR, Mildon A, Siekmans K, Main B, Macdonald C. An adequacy evaluation of a 10-year, four-country nutrition and health programme. International Journal of Epidemiology. 2010; 39(2):613-29.
16. Bini S, Cerri, C, Rigamonti, AE, Bertazzi, PA, Fiorini, G, Cella, SG. Pharmacoepidemiological data from drug dispensing charities as a measure of health patterns in a population not assisted by the Italian National Health Service. J Public Health Res. 2016;5(2):623.
17. Biswas T, Haider MM, Das Gupta R, Uddin J. Assessing the readiness of health facilities for diabetes and cardiovascular services in Bangladesh: a cross-sectional survey. BMJ Open. 2018;8(10):e022817.
18. Bradley, EH, Curry, LA, Taylor, LA, Pallas, SW, Talbert-Slagle, K, Yuan, C, Fox, A, Minhas, D, Ciccone, DK, Berg, D. Pérez-Escamilla, R. A model for scale up of family health innovations in low-income and middle-income settings: A mixed methods study. BMJ Open; 2(4): e000987.
19. Cancedda C, Davis, S. M, Dierberg, K.L, Lascher, J, Kelly, J. D, Barrie, M. B, Koroma, A.P, George, P, Kamara, A.A, Marsh, R, Sumbuya, M. S, Nutt, C. T, Scott, K.W, Thomas, E, Bollbach, K, Sesay, A, Barrie, A, Barrera, E, Barron, K, Welch, J, Bhadelia, N, Frankfurter, R. G, Dahl, O.M, Das, S, Rollins, R.E, Eustis, B, Schwartz, A, Pertile, P, Pavlopoulos, I, Mayfield, A, Marsh, R. H, Dibba, Y, Kloepper, D, Hall, A., Huster, K., Grady, M., Spray, K., Walton, D.A, Daboh, F., Nally, C., James, S., Warren, G.S., Chang, J., Drasher, M., Lamin, G., Bangura, S., Miller, A. C., Michaelis, A. P., McBain, R., Broadhurst, M. J., Murray, M., Richardson, E. T., Philip, T, Gottlieb, G. L, Mukherjee, J. S, Farmer, P. E. Strengthening health systems while responding to a health crisis: lessons learned by a nongovernmental organization during the Ebola virus disease epidemic in Sierra Leone. J Infect Dis. 2016;214(suppl 3):S153-s63.
20. Cantril C, Haylock, PJ. Patient navigation in the oncology care setting. Seminars in Oncology Nursing. 2013;29(2):76-90.
21. Carlozzi NE, Lange RT, French LM, Sander AM, Freedman J, Brickell TA. A latent content analysis of barriers and supports to healthcare: perspectives from caregivers of service members and veterans with military-related traumatic brain injury. J Head Trauma Rehabil. 2018;33(5):342-53.
22. Carlson LC, Hatcher KW, Tomberg L, Kabetu C, Ayala R, Burg RV. Inequitable access to timely cleft palate surgery in low- and middle-income countries. World Journal of Surgery. 2016;40(5):1047-52.
23. Carter, JG, Sherbon, BJ, Viney, IS. United Kingdom health research analyses and the benefits of shared data. Health Research Policy and Systems. 2016; 14(9). <https://health-policy-systems.biomedcentral.com/articles/10.1186/s12961-016-0116-1#citeas>.
24. Chandra-Mouli V, Lenz C, Adebayo E, Lang Lundgren I, Gomez Garbero L, Chatteriee S. A systematic review of the use of adolescent mystery clients in assessing the adolescent friendliness of health services in high, middle, and low-income countries. Glob Health Action. 2018;11(1):1536412.
25. Chowdhury AMR, Bhuiya, A, Chowdhury, ME, Rasheed, S, Hussain, Z, Chen, LC. The Bangladesh paradox: exceptional health achievement despite economic poverty. The Lancet. 2019;382(9906):1734-45.
26. Chu, K, Rosseel, P, Trelles, M, Gielis, P. Surgeons without borders: a brief history of surgery at médecins sans frontières. World Journal of Surgery. 2010;34(3):411-414.
27. Coe, C, Barlow, J. Supporting women with perinatal mental health problems: the role of the voluntary sector. Community Practitioner. 2013;86(2):23-27.
28. Colombini M, Ali SH, Watts C, Mayhew SH. One stop crisis centres: A policy analysis of the Malaysian response to intimate partner violence. Health Research Policy Systems. 2011;9:25.
29. Corbin JH, Mittelmark MB, Lie GT. Scaling-up and rooting-down: a case study of North-South partnerships for health from Tanzania. Global Health Action. 2012;5:18369.
30. Cunningham C, Wensley, R, Blacker, D, Bache, J, Stonier, C. Occupational therapy to facilitate physical activity and enhance quality of life for individuals with complex neurodisability. British Journal of Occupational Therapy. 2012;75:106–10.
31. Dasgupta, J. Ten years of negotiating rights around maternal health in Uttar Pradesh, India. BMC International Health and Human Rights. 2011;11:11.
32. Davis SL, Goedel WC, Emerson J, Guven BS. Punitive laws, key population size estimates, and Global AIDS Response Progress Reports: an ecological study of 154 countries. Journal of the International AIDS Society. 2017;20(1).
33. Deboutte D, O'Dempsey, T, Mann, G, Faragher, B. Cost-effectiveness of caesarean sections in a post-conflict environment: a case study of Bunia, Democratic Republic of the Congo. Disasters. 2013;37:S105-20.
34. Deleye C, Lang A. Maternal health development programs: comparing priorities of bilateral and private donors. BMC Int Health Hum Rights. 2014;14.
35. Descoteaux N, Chagnon V, Di Dong X, Ellemo E, Hamelin A, Juste E, Laplante X, Miron A, Morency P, Samuel K, Charles D, Hunt M. Expanding the Haitian rehabilitation workforce: employment situation and perceptions of graduates from three rehabilitation technician training programs. Disabil Rehabil. 2018;40(10):1227-36.
36. Diggle E, Welsch W, Sullivan R, Alkema G, Warsame A, Wafai M, Jasem M, Ekzayez A, Cummings R, Patel P. The role of public health information in assistance to populations living in opposition and contested areas of Syria, 2012-2014. Conflict and Health. 2017;11:33.
37. Duchenko, A, Deshko, T, Braga, M. Crisis management by HIV/AIDS non-governmental organisations in the post-Euromaidan Ukraine led to opening new horizons. Drugs and Alcohol Today. 2017;17(3):149-156.
38. Dunn J, Herron, L, Adams, C, Chambers, S. Engaging NGOs in national cancer-control efforts. The Lancet Oncology. 2013;14(11):1044-6.
39. Dunn JT, Lesyna K, Zaret A. The role of human rights litigation in improving access to reproductive health care and achieving reductions in maternal mortality. BMC Pregnancy and Childbirth. 2017;17(2):1-13.
40. Ekirapa A, Mgomella GS, Kyobutungi C. Civil society organizations: Capacity to address the needs of the urban poor in Nairobi. Journal of Public Health Policy. 2012; 33(4): 404-422.
41. El Arifeen S, Christou A, Reichenbach L, Osman, FA, Azad K, Islam KS, Ahmed F, Perry HB, Peters DH. Community-based approaches and partnerships: innovations in health-service delivery in Bangladesh. The Lancet. 2013;382(9909):P2012-26.
42. Elmontsri, M, Almashrafi, A, Dubois, E, Banarsee, R, Majeed, A. Improving patient safety in Libya: insights from a British health system perspective. International Journal of Health Care Quality Assurance. 2018;31(3):237-248.
43. Engelman A, Ivey SL, Tseng W, Dahrouge D, Brune J, Neuhauser L. Responding to the deaf in disasters: establishing the need for systematic training for state-level emergency management agencies and community organizations. BMC Health Services Research. 2013;13(1):1-10.
44. Evensen JV, Stokke, K. United Against HIV/AIDS? Politics Of Local Governance In HIV/AIDS Treatment In Lusikisiki, South Africa. Journal of Southern African Studies. 2010;36(1):151-67.
45. Fast I, Sorensen K, Brand H, Suggs LS. Social media for public health: an exploratory policy analysis. Eur J Public Health. 2015;25(1):162-6.
46. Fehling M, Nelson BD, Ahn R, Eckardt M, Tiernan M, Purcell G, El-Bashir A, Burke TF. Development of a community-based maternal, newborn and child emergency training package in South Sudan. Public Health. 2013;127(9):797-805.
47. Fiorini G, Cerri C, Bini S, Rigamonti AE, Perlini S, Marazzi N, Sartorio A, Cella SG. The burden of chronic noncommunicable diseases in undocumented migrants: a 1-year survey of drugs dispensation by a non-governmental organization in Italy. Public Health. 2016;141:26-31.
48. Flitcroft K, Gillespie J, Salkeld G, Carter S, Trevena L. Getting evidence into policy: the need for deliberative strategies? Social Science and Medicine. 2011; 72(7): 1039-1046.
49. Franco JR, Cecchi G, Priotto G, Paone M, Diarra A, Grout L, Simarro PP, Zhao W, Argaw D. Monitoring the elimination of human African trypanosomiasis: Update to 2016. PLoS Negl Trop Dis. 2018;12(12):e0006890.
50. Frost, A, Wilkinson, M, Boyle, P, Patel, P, Sullivan, R. An assessment of the barriers to accessing the Basic Package of Health Services (BPHS) in Afghanistan: was the BPHS a success? Globalization and Health. 2016;12:11.
51. Furey A, Rourke, J, Larsen, H. Building the capacity to manage orthopaedic trauma after a catastrophe in a low-income country. Journal of Orthopaedic Trauma. 2015;29:S14-6.
52. Gaber J. Seeing the community's perspective through multiple emic and etic vistas. Health Promot Int. 2017;32(6):1025-33.
53. George AS, Mohan D, Gupta J, LeFevre AE, Balakrishnan S, Ved S, Khanna, R. Can community action improve equity for maternal health and how does it do so? Research findings from Gujarat, India. International Journal for Equity in Health. 2018;17(1):1-11.
54. George AS, Branchini C. Principles and processes behind promoting awareness of rights for quality maternal care services: a synthesis of stakeholder experiences and implementation factors. BMC Pregnancy and Childbirth. 2017;17:15.
55. Gherman L, Pogonet V, Soltan V, Isac V. Palliative care in Moldova. Journal of Pain and Symptom Management. 2018;55(2):S55-S8.
56. Goldman J, Meuser J, Lawrie L, Rogers J, Reeves S. Interprofessional primary care protocols: A strategy to promote an evidence-based approach to teamwork and the delivery of care. Journal of Interprofessional Care. 2010;24(6):653-665.
57. Gómez E, Harris J. Political repression, civil society and the politics of responding to AIDS in the BRICS nations. Health Policy and Planning. 2016;31(1):56-66.
58. Guinness L. What can transaction costs tell us about governance in the delivery of large scale HIV prevention programmes in southern India? Social Science & Medicine. 2011;72(12):1939-47.
59. Gurung A, Narayanan P, Prabhakar P, Das A, Ranebennur V, Tucker S, Narayana L, Radha R, Prakash K, Touthang J, Sono CZ, Wi T, Morineau G, Neilsen G. Large-scale STI services in Avahan improve utilization and treatment seeking behaviour amongst high-risk groups in India: an analysis of clinical records from six states. BMC Public Health. 2011;11(Suppl 6):S10.
60. Gutnik L, Dieleman J, Dare AJ, Ramos MS, Riviello R, Meara JG, Yamey G, Shrime MG. Funding allocation to surgery in low and middle-income countries: a retrospective analysis of contributions from the USA. BMJ Open. 2015;5(11):e008780.
61. Ha BTT, Frizen S, Thi LM, Duong DTT, Duc DM. Policy processes underpinning universal health insurance in Vietnam. Glob Health Action. 2014;7.
62. Haakenstad A, Johnson E, Graves C, Olivier, J, Duff J, Dieleman JL. Estimating the development assistance for health provided to faith-based organizations, 1990–2013. PLOS ONE. 2015;10(6):e0128389.
63. Hafeez S, Singhera M, Huddart R. Exploration of the treatment challenges in men with intellectual difficulties and testicular cancer as seen in Down syndrome: single centre experience. BMC Medicine. 2015;13(1):1-7.
64. Hall MA. Access to care provided by better safety net systems for the uninsured: measuring and conceptualizing adequacy. Medical Care Research and Review. 2011;68(4):441-461.
65. Hannah EN. NGOs and the European Union: Examining the power of epistemes in the EC's TRIPS and access to medicines negotiations. Journal of Civil Society. 2011;7(2):179-206.
66. Herring B, Gaskin D, Zare H, Anderson G. Comparing the Value of Nonprofit Hospitals’ Tax Exemption to Their Community Benefits. Inquiry. 552018.
67. Huaynoca S, Chandra-Mouli V, Yaqub Jr. N, Denno DM. Scaling up comprehensive sexuality education in Nigeria: from national policy to nationwide application. Sex Education. 2014;14(2):191-209.
68. Humphries D, Gomez L, Hartwig K. Sustainability of NGO capacity building in southern Africa: successes and opportunities. International Journal of Health Planning and Management. 2011;26(2):e85-101.
69. Hunt MR. Establishing moral bearings: ethics and expatriate health care professionals in humanitarian work. Disasters. 2011;35(3):606-22.
70. Iliffe S, Davies SL, Gordon AL, Schneider J, Dening T, Bowman C, Gage H, Martin FC, Gladman JR, Victor C, Meyer J, Goodman C. Provision of NHS generalist and specialist services to care homes in England: review of surveys. Primary Health Care Res and Development. 2016;17(2):122-37.
71. Ince P, Haddock G, Tai S. A systematic review of the implementation of recommended psychological interventions for schizophrenia: Rates, barriers, and improvement strategies. Psychol Psychother. 2016;89(3):324-50.
72. Ingrosso L, Schmidt T, Sherally J, Dembech M, Montes SB, Machado RS, Annunziata G, Rezza, G Severoni S. A desk review on institutional and non-institutional organizations active in the field of migrant's health in the WHO European Region. Annali dell'Istituto Superiore di Sanita. 2015;51(4):313-320.
73. Jacobs B, Thome JM, Overtoom R, Sam SO, Indermuhle L, Price N. From public to private and back again: sustaining a high service-delivery level during transition of management authority: a Cambodia case study. Health Policy and Planning. 2010;25(3):197-208.
74. Kakalou E, Riza E, Chalikias M, Voudouri N, Vetsika A, Tsiamis C, Choursoglou S, Terzidis A, Karamagioli E, Antypas T, Pikoulis E. Demographic and clinical characteristics of refugees seeking primary healthcare services in Greece in the period 2015–2016: a descriptive study. International Health. 2018;10(6):421-9.
75. Källander K, Tibenderana JK, Akpogheneta OJ, Strachan DL, Hill Z, Asbroek, AHAT, Conteh L, Kirkwood BR, Meek SR. Mobile health (mhealth) approaches and lessons for increased performance and retention of community health workers in low and middle-income countries: a review. Journal of Medical Internet Research. 2013;15(1):e17.
76. Kara H, Arvidson M. To what extent can evaluation frameworks help NGOs to address health inequalities caused by social exclusion? Perspectives in Public Health. 2015;135(4);191-196.
77. Kelly RP, Burke J, Waddell S, Lachance L. Increasing opportunities for health in a southeast Michigan community through local policy change. Health Promotion Practice. 2018;20(1):116–27.
78. Kempers J, Ketting E, Chandra-Mouli V, Raudsepp T. The success factors of scaling-up Estonian sexual and reproductive health youth clinic network - from a grassroots initiative to a national programme 1991–2013. Reproductive Health. 2015;12(2):PMC4298051.
79. Kilic B, Kalaca S, Unal B, Phillimore P, Zaman S. Health policy analysis for prevention and control of cardiovascular diseases and diabetes mellitus in Turkey. International Journal of Public Health. 2015;60:S47-53.
80. Knijn T, Slabbert M. Transferring HIV/AIDS related healthcare from non-governmental organizations to the public healthcare system in South Africa: opportunities and challenges. Social Policy and Administration. 2012;46(6):636-653.
81. Kohli A, Makambo MT, Ramazani P, Zahiga I, Mbika B, Safari O, Bachunguye R, Mirindi J, Glass N. A Congolese community-based health program for survivors of sexual violence. Conflict and Health. 2012;6(1):6.
82. Koivusalo M, Mackintosh M. Commercial influence and global nongovernmental public action in health and pharmaceutical policies. International Journal of Health Services. 2011;41(3):539-63.
83. Kraak VI, Harrigan PB, Lawrence M, Harrison PJ, Jackson MA, Swinburn B. Balancing the benefits and risks of public-private partnerships to address the global double burden of malnutrition. Public Health Nutrition. 2012;15(3):503-17.
84. Larson E, O'Bra H, Brown JW, Mbengashe T, Klausner JD. Supporting the massive scale-up of antiretroviral therapy: the evolution of PEPFAR-supported treatment facilities in South Africa, 2005-2009. BMC Public Health. 2012;12:173.
85. Lavoie J, Varcoe C, Wathen CN, Ford-Gilboe M, Browne AJ. Sentinels of inequity: examining policy requirements for equity-oriented primary healthcare. BMC Health Services Research. 2018;18(1):1-12.
86. Libman K, Freudenberg N, Sanders D, Puoane T, Tsolekile L. The role of urban food policy in preventing diet-related non-communicable diseases in Cape Town and New York. Public Health. 2015;129(4):327-335.
87. Lindgren TG, Deutsch K, Schell E, Bvumbwe A, Hart KB, Laviwa J, Rankin SH. Using mobile clinics to deliver HIV testing and other basic health services in rural Malawi. Rural Remote Health. 2011;11(2):1682.
88. Lipsky AB. Evaluating the strength of faith: potential comparative advantages of faith-based organizations providing health services in sub-Saharan Africa. Public Administration and Development. 2011;31(1):25-36.
89. Llano R, Kanamori S, Kunii O, Mori R, Takei T, Sasaki H, Nakamura Y, Kurokawa K, Hai Y, Chen L, Takemi K, Shibuya K. Re-invigorating Japan's commitment to global health: challenges and opportunities. The Lancet. 2011;378(9798):1255-1264
90. London L, Fick N, Tram KH, Stuttaford M. Filling the gap: a learning network for health an human rights in the Western Cape, South Africa. Health Hum Rights. 2012;14(1):E88-105.
91. Loyer AB, Ali M, Loyer D. New politics, an opportunity for maternal health advancement in eastern myanmar: an integrative review. Journal of Health, population and nutrition. 2014;32(3):471-85.
92. Lyness SM, McCambridge J. The alcohol industry, charities and policy influence in the UK. European Journal of Public Health. 2014;24(4):557-61.
93. MacKenzie R, Collin J. 'A preferred consultant and partner to the Royal Government, NGOs, and the community': British American Tobacco's access to policy-makers in Cambodia. Glob Public Health. 2017;12(4):432-48.
94. Maleka EN. Monitoring and evaluation of sport-based HIV/AIDS awareness programmes: Strengthening outcome indicators. SAHARA J. 2017;14(1):1-21.
95. Marsden J, Eastwood, B, Jones, H, Bradbury, C, Hickman, M, Knight, J, Randhawa, K, White, M, the National Drug Treatment Monitoring System Outcomes Study Group. Risk adjustment of heroin treatment outcomes for comparative performance assessment in England. Addiction. 2012;107(12):2161-72.
96. Martos AJ, Wilson PA, Meyer IH. Lesbian, gay, bisexual, and transgender (LGBT) health services in the United States: Origins, evolution, and contemporary landscape. PLoS ONE. 2017;12(7): e0180544.
97. McGinn, T, Casey, SE. Why don’t humanitarian organizations provide safe abortion services? Conflict and Health. 2016;10: 8.
98. Mercer MA, Thompson, SM, de Araujo, RM. The role of international NGOs in health systems strengthening: the case of Timor-Leste. International Journal of Health Services. 2014;44(2):323-35.
99. Moon S, Sridhar D, Pate MA, Jha AK, Clinton C, Delaunay S, Edwin V, Fallah M, Fidler DP, Garrett L, Goosby E, Gostin LO, Heymann DL, Lee K, Leung GM, Morrison JS, Saavedra J, Tanner M, Leigh JA, Hawkins B, Woskie LR, Piot P. Will Ebola change the game? Ten essential reforms before the next pandemic. The report of the Harvard-LSHTM Independent Panel on the Global Response to Ebola. The Lancet. 2015;386(10009):2204-2221.
100. Moscou K, Kohler JC. Matching safety to access: global actors and pharmacogovernance in Kenya- a case study. Globalization and Health. 2017;13(16):20.
101. Mprah WK, Anafi P, Sekyere FO. Does disability matter? Disability in sexual and reproductive health policies and research in Ghana. Int Q Community Health Educ. 2014;35(1):21-35.
102. MR BL, Hulme J, Johnson K. Payday, ponchos, and promotions: a qualitative analysis of perspectives from non-governmental organization programme managers on community health worker motivation and incentives. Hum Resour Health. 2014;12:66.
103. Murdie A. Scrambling for contact: The determinants of inter-NGO cooperation in non-Western countries. The Review of International Organizations. 2014;9(3):309-31.
104. Ng-Kamstra JS, Riesel JN, Arya S, Weston B, Kreutzer T, Meara JG, Shrime MG. Surgical Non-governmental Organizations: Global Surgery's Unknown Nonprofit Sector. World Journal of Surgery. 2016;40(8):1823-41.
105. Nikpay SS, Ayanian JZ. Hospital Charity Care--Effects of New Community-Benefit Requirements. N Engl J Med. 2015;373(18):1687-90.
106. Novakowski AK, Garcia-Gonzalez P, Wrigglesworth M, Stergachis A. A pharmacovigilance system for treatment access and medical donation programs: the Max Foundation experience. Global Health. 2018;14(1):76.
107. Odwe G, Gray K, Kyarimpa A, Obare F, Nagendi G. Introduction of subcutaneous depot Medroxyprogesterone Acetate (DMPA-SC) injectable contraception at facility and community levels: pilot results from 4 districts of Uganda. Glob Health Sci Pract. 2018;6(4):711-22.
108. Olivier de Sardan JP, Diarra A, Kone FY, Yaogo M, Zerbo R. Local sustainability and scaling up for user fee exemptions: medical NGOs vis-a-vis health systems. BMC Health Serv Res. 2015;15 Suppl 3:S5.
109. Patel P, Roberts B, Conteh L, Guy S, Lee-Jones L. A review of global mechanisms for tracking official development assistance for health in countries affected by armed conflict. Health Policy. 2011;100(2-3):116-24.
110. Penas Defago MA, Moran Faundes, JM. Conservative litigation against sexual and reproductive health policies in Argentina. Reproductive Health Matters. 2014;22(44):82-90.
111. Perry L, Malkin R. Effectiveness of medical equipment donations to improve health systems: How much medical equipment is broken in the developing world? Medical and Biological Engineering and Computing. 2011; 49(7):719-722.
112. Pharoah C, Harrow J. A legacy for the nation's health – the challenges faced by UK health charities in legacy funding. Journal of Communication in Healthcare. 2011;4(1):13-26.
113. Piotrowicz M, Cianciara D. Associations and foundations in the field of health care and their role in the health system of Poland. Przegla̧d epidemiologiczny. 2013;67(1):63-68, 145-149.
114. Piotrowicz M, Cianciara D. The role of non-governmental organizations in the social and the health system. Przegla̧d epidemiologiczny. 2013;67(1):151-155.
115. Poenaru D. Getting the job done: analysis of the impact and effectiveness of the SmileTrain program in alleviating the global burden of cleft disease. World Journal of Surgery. 2013;37(7):1562-70.
116. Price E, Baker R, Krause J, Keen C. Organisation of services for people with cardiovascular disorders in primary care: transfer to primary care or to specialist-generalist multidisciplinary teams? BMC Family Practice. 2014;22(15):158.
117. Qirbi N, Ismail, SA. Health system functionality in a low-income country in the midst of conflict: the case of Yemen. Health Policy and Planning. 2017;32(6):911-22.
118. Reeve J, Cooper L, Harrington S, Rosbottom P, Watkins J. Developing, delivering and evaluating primary mental health care: the co-production of a new complex intervention. BMC Health Services Research. 2016;16:13.
119. Richardson E, Birn A.E. Sexual and reproductive health and rights in Latin America: An analysis of trends, commitments and achievements. Reproductive Health Matters. 2011;19(38):183-196.
120. Robert E, Ridde V. Global health actors no longer in favor of user fees: a documentary study. Global Health. 2013;26(9):29.
121. Ron Levey I, Wang W. Unravelling the quality of HIV counselling and testing services in the private and public sectors in Zambia. Health Policy Plan. 2014;29(Suppl 1):i30-7.
122. Rosenquist R, Golichenko O, Roosen T, Ravenscroft, J. A critical player: the role of civil society in achieving universal health coverage. Global Health Governance. 2013;6(2). <http://blogs.shu.edu/wp-content/blogs.dir/109/files/2014/02/GHGJ_62_100-105_ROSENQUIST_ET_AL.pdf>.
123. Round J, Jones, L, Morris, S. Estimating the cost of caring for people with cancer at the end of life: A modelling study. Palliative Medicine. 2015;29(10):899-907.
124. Ruckstuhl L, Lengeler C, Moyen JM, Garro H, Allan R. Malaria case management by community health workers in the Central African Republic from 2009–2014: overcoming challenges of access and instability due to conflict. Malar J. 2017;16(1):388.
125. Sandoval C, Cáceres CF. Influence of health rights discourses and community organizing on equitable access to health: the case of HIV, tuberculosis and cancer in Peru. Global Health. 2013;9:23.
126. Sasaki H, Bouesseau MC, Marston J, Mori R. A scoping review of palliative care for children in low- and middle-income countries. BMC Palliative Care. 2017;16(60): PMID29178866.
127. Shah NK. Corporate philanthropy and conflicts of interest in public health: ExxonMobil, Equatorial Guinea, and malaria. Journal of Public Health Policy. 2013; 34(1): 121-136.
128. Sherwood J, Sharp A, Honermann B, Horrigan C, Chatterjee M, Jones A, Cooney C, Millett G. Mapping the impact of the expanded Mexico City Policy for HIV/ family planning service integration in PEPFAR-supported countries: a risk index. BMC Public Health. 2018;18(1):1116.
129. Shipman KE, Doyle A, Arden H, Jones T, Gittoes NJ. Development of fracture liaison services: what have we learned? Injury. 2017;48:S4-S9.
130. Shohel MMC, Howes AJ. The relevance of formal and nonformal primary education in relation to health, well-being and environmental awareness: Bangladeshi pupils' perspectives in the rural contexts. Int J Qual Stud Health Well-being. 2018;13(1):1554022.
131. Slosar JP, Repenshek MF, Bedford E. Catholic Identity and Charity Care in the Era of Health Reform. HEC Forum. 2013;25(2):111-26.
132. Smith J, Mallouris C, Lee K, Alfvén T. The role of civil society organizations in monitoring the global AIDS response. AIDS and Behavior. 2017;21:44-50.
133. Sodhi C, Singh P. Health service system in transition: an assessment of the influence of the British and US healthcare systems on the evolution of health services in India. International Journal of Health Governance. 2016;21(4):204-21.
134. Sparrow A, Almilaji K, Tajaldin B, Teodoro N, Langton P. Cholera in the time of war: implications of weak surveillance in Syria for the WHO's preparedness-a comparison of two monitoring systems. BMJ Global Helath. 2016;1(3):e000029.
135. Steinhardt LC, Aman I, Pakzad I, Kumar B, Singh LP, Peters DH. Removing user fees for basic health services: a pilot study and national roll-out in Afghanistan. Health Policy and Planning. 2011;26:ii92-ii103.
136. Steyn, PS, Cordero JP, Gichangi P, Smit JA, Nkole T, Kiarie J, Temmerman M. Participatory approaches involving community and healthcare providers in family planning/contraceptive information and service provision: A scoping review. Reproductive Health. 2016;13(1):88.
137. Stubbe Østergaard L, Norredam M, Mock-Munoz De Luna C, Blair M, Goldfeld S, Hjern A. Restricted health care entitlements for child migrants in Europe and Australia. European Journal of Public Health. 2017;27(5):869-873
138. Subedi R, Jahan I, Baatsen P. Factors Influencing Modern Contraceptive Use among Adolescents in Nepal. J Nepal Health Res Counc. 2018;16(3):251-6.
139. Sulzbach S, De S, Wang W. The private sector role in HIV/AIDS in the context of an expanded global response: Expenditure trends in five sub-Saharan African countries. Health Policy and Planning. 2011;26(Suppl. 1):i72-i84.
140. Taylor CB, Stevenson M, Jan S, Liu B, Tall G, Middleton PM, Fitzharris M, Myburgh J. An investigation into the cost, coverage and activities of helicopter emergency medical services in the state of New South Wales, Australia. Injury. 2011;42(10):1088-94.
141. Tinker A, Parker R, Lord D, Grear K. Advancing newborn health: the Saving Newborn Lives initiative. Global Public Health. 2010;5(1):28-47.
142. Titmarsh S. Vision for Mental Health needs financial commitment. Progress in Neurology and Psychiatry. 2010;14(1):25.
143. Todrys KW, Amon JJ, Malembeka G, Clayton M. Imprisoned and imperiled: access to HIV and TB prevention and treatment, and denial of human rights, in Zambian prisons. Journal of the International AIDS Society. 2011;14:8.
144. Tremblay C, Coulombe V, Briand C. Users’ involvement in mental health services: programme logic model of an innovative initiative in integrated care. International Journal of Mental Health Systems. 2017;11(1):9.
145. Tynan A, Vallely A, Kelly A, Law G, Millan J, Siba P, Kaldor J, Hill PS. Vasectomy as a proxy: extrapolating health system lessons to male circumcision as an HIV prevention strategy in Papua New Guinea. BMC Health Services Research. 2012;12(1):1-12.
146. Ui S, Heng L, Yatsuya H, Kawaguichi L, Akashi H, Aoyana A. Strengthening community participation at health centers in rural Cambodia: role of local non-governmental organizations (NGOs). Critical Public Health. 2010;20(1):97-115.
147. Verma R, Shekhar A, Khobragade S, Adhikary R, George B, Ramesh BM, Ranebennur V, Mondal S, Patra RK, Srinivasan S, Vijayaraman A, Paul SR, Bohidar N. Scale-up and coverage of Avahan: a large-scale HIV-prevention programme among female sex workers and men who have sex with men in four Indian states. Sexually Transmitted Infections. 2010;86:i76-82.
148. Vogus A, Graff K. PEPFAR transitions to country ownership: review of past donor transitions and application of lessons learned to the Eastern Caribbean. Global Health-Science and Practice. 2015;3(2):274-286.
149. Walton J. The role of non-governmental organizations in vaccine development and delivery. International Journal of Health Governance. 2017;22(3):152-160.
150. Wang EA, Hong CS, Samuels L, Shavit S, Sandersa R, Kushel M. Transitions clinic: creating a community-based model of health care for recently released California prisoners. Public Health Reports. 2010;125(2):171-177.
151. Wendland A, Ehmsen BK, Lenskjold V, Astrup BS, Mohr M, Williams CJ, Cowan, SA. Undocumented migrant women in Denmark have inadequate access to pregnancy screening and have a higher prevalence Hepatitis B virus infection compared to documented migrants in Denmark: a prevalence study. BMC Public Health. 162016.
152. Wu AW, Hwang S, Weston CM, Ibe C, Boonyasai RT, Bone L, Basu L, Lief I, Gentry J, Purnell L, Rosenblum M, Baltimore CONNECT Study Team. Baltimore CONNECT: A randomized trial to build partnership between community organizations and a local health system. Prog Community Health Partnersh. 2018;12(3):297-306.
153. Xu T. Chinese Anti-Cancer Association as a non-governmental organization undertakes systematic cancer prevention work in China. Chinese Journal of Cancer Research. 2015;27(4):423-427.
154. Yassin N, Taha AA, Ghantous Z, Atoui MM, Forgione F. Evaluating a mental health program for Palestinian refugees in Lebanon. J Immigr Minor Health. 2018;20(2):388-98.
155. Zabdyr-Jamróz M. Voluntary (NGO) Sector’s Involvement in Health Promotion for Older Population in Europe. Epidemiology biostatistics and public health. 2017;14(2):e12420-2.
156. Zaidi S, Mayhew SH, Palmer N. Bureaucrats as purchasers of health services: limitations of the public sector for contracting. Public administration and development. 2011;31:135-48.

## Articles which only used NGO data as a contextual or corroboratory reference (n=82)

1. Adisa R, Olajide OO, Fakeye TO. Social support, treatment adherence and outcome among hypertensive and type 2 diabetes patients in ambulatory care settings in southwestern Nigeria. Ghana Med J. 2017;51(2):64-77.
2. Albahari A, Schiltz CH. A qualitative analysis of the spontaneous volunteer response to the 2013 Sudan floods: changing the paradigm. Prehospital and Disaster Medicine. 2017;32(3):240-8.
3. Aldridge MD, Schlesinger M, Barry CL, Morrison RS, McCorkle R, Hurzeler R, Bradley EH. National hospice survey results: for-profit status, community engagement, and service. JAMA Intern Med. 2014;174(4):500-6.
4. Alhassan RK, Nketiah-Amponsah E, Spieker N, Arhinful DK, Rinke de Wit TF. Assessing the Impact of Community Engagement Interventions on Health Worker Motivation and Experiences with Clients in Primary Health Facilities in Ghana: A Randomized Cluster Trial. PLoS One. 2016;11(7):e0158541.
5. AlKhaldi M, Abed Y, Pfeiffer C, Haj-Yahia S, Alkaiyat A, Tanner M. Assessing policy-makers’, academics’ and experts’ satisfaction with the performance of the Palestinian health research system: a qualitative study. Health Research Policy and Systems. 2018;16(1):1-11.
6. Amuasi JH, Diap G, Nguah SB, Karikari P, Boakye I, Jambai A, Lahai WK, Louie KS, Kiechel JR. Access to artemisinin-combination therapy (ACT) and other anti-malarials: national policy and markets in Sierra Leone. PLoS One. 2012;7(10):e47733.
7. Aseyo RE, Mumma J, Scott K, Nelima D, Davis E, Baker KK, et al. Realities and experiences of community health volunteers as agents for behaviour change: evidence from an informal urban settlement in Kisumu, Kenya. Hum Resour Health. 2018;16(1):53.
8. Asikhia OA, Mohangi K. A case study of school support and the psychological, emotional and behavioural consequences of HIV and AIDS on adolescents. Sahara j. 2015;12:123-33.
9. Baird E, Williams, ACC, Hearn, L, Amris, K. Interventions for treating persistent pain in survivors of torture. Cochrane Database Systematic Reviews. 2017;8:Cd012051.
10. Basu S, Andrews, J, Kishore, S, Panjabi, R, Stuckler, D. Comparative performance of private and public healthcare systems in low- and middle-income countries: a systematic review. PLoS Medicine. 2012;9(6):e1001244.
11. Bedford Russell AR, Passant M, Kitt H. Engaging children and parents in service design and delivery. Archives of Disease and Childhood. 2014;99(12):1158-62.
12. Berry SA, Fleishman JA, Yehia BR, Cheever LW, Hauck H, Korthuis PT, Mathews WC, Keruly J, Nijhawan AE, Agwu AL, Somboonwit C, Moore RD, Gebo KA, HIV Research Network. Healthcare coverage for HIV provider visits before and after implementation of the Affordable Care Act. Clin Infect Dis. 2016;63(3):387-95.
13. Blitz BK, D’Angelo A, Kofman E, Montagna N. Health challenges in refugee reception: Dateline Europe 2016. International Journal of Environmental Research and Public Health. 2017;14(12):e1484
14. Bobrovitz N, Heneghan C, Onakpoya I, Fletcher B, Collins D, Tompson A, Lee J, Nunan D, Fisher R, Scott B, O'Sullivan J, Van Hecke O, Nicholson BD, Stevens S, Roberts N, Mahtani KR. Medications that reduce emergency hospital admissions: an overview of systematic reviews and prioritisation of treatments. BMC Med. 2018;16(1):115.
15. Boivin, A, Lehoux, P, Lacombe, R, Burgers, J, Grol, R. Involving patients in setting priorities for healthcare improvement: a cluster randomized trial. Implementation Science. 2014;9(1):24
16. Cailhol J, Craveiro I, Madede T, Makoa E, Mathole T, Parsons AN, Van Leemput L, Biesma R, Brugha R, Chilundo B, Lehmann U, Dussault G, Van Damme W, Sanders D. Analysis of human resources for health strategies and policies in 5 countries in Sub-Saharan Africa, in response to GFATM and PEPFAR-funded HIV-activities. Globalization and Health. 2013;9:14.
17. Chaufan C, Constantino S, Davis M. ‘It's a full time job being poor’: understanding barriers to diabetes prevention in immigrant communities in the USA. Critical Public Health. 2012;22(2):147-58.
18. Chinyama MJ, MacLachlan M, McVeigh J, Huss T, Gawamadzi S. An analysis of the extent of social inclusion and equity consideration in Malawi's national HIV and AIDS policy review process. International Journal of Health Policy and Management. 2018;7(4):297-307.
19. Chuah FLH, Tan ST, Yeo J, Legido-Quigley H. The health needs and access barriers among refugees and asylum-seekers in Malaysia: a qualitative study. Int J Equity Health. 2018;17(120).
20. Clemans-Cope L, Long SK, Coughlin TA, Yemane A, Resnick D. The expansion of Medicaid coverage under the ACA: implications for health care access, use, and spending for vulnerable low-income adults. Inquiry. 2013;50(2):135-49.
21. Cohen JE, Amon JJ. Lead poisoning in China: a health and human rights crisis. Health Hum Rights. 2012;14(2):74-86.
22. Cohen W, McCartney E, Crampin L. 22q11 deletion syndrome: Parents' and children's experiences of educational and healthcare provision in the United Kingdom. undefined. 2017;21:142-52.
23. Crosbie E, Thomson G. Regulatory chills: tobacco industry legal threats and the politics of tobacco standardised packaging in New Zealand. N Z Med J. 2018;131(1473):25-41.
24. Cuadra CB. Right of access to health care for undocumented migrants in EU: a comparative study of national policies. European Journal of Public Health. 2012;22(2):267-271.
25. Daykin N, de Viggiani N, Moriarty Y, Pilkington P. Music-making for health and wellbeing in youth justice settings: mediated affordances and the impact of context and social relations. Sociology of Health & Illness. 2017;39(6):941-958.
26. Derderian K, Schockaert L. Can aid switch gears to respond to sudden forced displacement? The case of Haut-Uele, DRC. Refuge: Canada's Periodical on Refugees. 2011;27(1):16-23.
27. Devillé W, Greacen T, Bogic M, Dauvrin M, Dias S, Gaddini A, Jensen N.K, Karamanidou C, Kluge U, Mertaniemi R, Riera R.P, Sárváry A, Soares J.J, Stankunas M, Strassmayr C, Welbel M, Priebe S. Health care for immigrants in Europe: is there still consensus among country experts about principles of good practice? A Delphi study. BMC Public Health. 2011;13(11):699.
28. Edoka I, Ensor T, McPake B, Amara R, Tseng FM, Edem-Hotah J. Free health care for under-fives, expectant and recent mothers? Evaluating the impact of Sierra Leone's free health care initiative. Health Econ Rev. 2016;6(1):19.
29. Ewen JE. Using the Venom of a Snake for an Antidote: exploring strategies and services for sex workers in preparation for the World Cup 2014. Perspect Public Health. 2015;135(4):197-203.
30. Floyd K, Fitzpatrick C, Pantoja A, Raviglione M. Domestic and donor financing for tuberculosis care and control in low-income and middle-income countries: An analysis of trends, 2002-11, and requirements to meet 2015 targets. The Lancet Global Health. 2013; 1(2):e105-e115.
31. Gibbs A, Mushinga M, Crone ET, Willan S, Mannell J. How do national strategic plans for HIV and AIDS in southern and eastern Africa address gender-based violence? A women's rights perspective. Health Hum Rights. 2012;14(2):10-20.
32. Giri A, Khatiwada P, Shrestha B, Chettri RK. Perceptions of government knowledge and control over contributions of aid organizations and INGOs to health in Nepal: a qualitative study. Global Health. 2013;9:1.
33. Glueckert LN, Redden D, Thompson MA, Haque A, Gray SH, Locke J, Eckhoff DE, Fouad M, DuBay DA. What liver transplant outcomes can be expected in the uninsured who become insured via the Affordable Care Act? Am J Transplant. 2013;13(6):1533-40.
34. Goldzweig IA, Schlundt DG, Moore WE, Smith PE, Zoorob RJ, Levine RS. An academic, business, and community alliance to promote evidence-based public health policy: the case of primary seat belt legislation. J Health Care Poor Underserved. 2013;24(3):1364-77.
35. Gomez EJ. Crafting AIDS policy in Brazil and Russia: State-civil societal ties, institutionalised morals, and foreign policy aspiration. Glob Public Health. 2016;11(9):1148-68.
36. Gordon S. The military physician and contested medical humanitarianism: a dueling identity? Soc Sci Med. 2014;120:421-9.
37. Grund JP, Latypov A, Harris M. Breaking worse: the emergence of krokodil and excessive injuries among people who inject drugs in Eurasia. Int J Drug Policy. 2013;24(4):265-74.
38. Haghparast-Bidgoli H, Shaha SK, Kuddus A, Chowdhury MAR, Jennings H, Ahmed N, Morrison J, Akter K, Nahar B, Nahar T, King C, Skordis-Worrall J, Batura N, Khan JA, Mansaray A, Hunter R, Khan AKA, Costello A, Azad K, Fottrell E. Protocol of economic evaluation and equity impact analysis of mHealth and community groups for prevention and control of diabetes in rural Bangladesh in a three-arm cluster randomised controlled trial. BMJ Open. 2018;8(8):e022035.
39. Husaini S, Maman RI. Stakeholders' misbehavior conduct in HIV/AIDS mitigations in the era of Indonesian decentralization and democracy. Indian Journal of Public Health Research & Development. 2017;8(4):335-339.
40. Iyer IR, Mackall J. Patient preferences regarding device reuse and potential of devices for reuse - a study in a veteran population. Indian Pacing Electrophysiol J. 2013;13(3):101-8.
41. Kaji A, Thi SS, Smith T, Charunwatthana P, Nosten FH. Challenges in tackling tuberculosis on the Thai-Myanmar border: findings from a qualitative study with health professionals. Bmc Health Services Research. 2015;15(9):464.
42. Khalily MT. Mental health problems in Pakistani society as a consequence of violence and trauma: a case for better integration of care. Interntional Journal of Integrated Care. 2011;11:e128.
43. Khan MA, Xiaoying J, Kanwal N. Armed conflict in the federally administered tribal areas of Pakistan and the role of NGOs in restoring health services. Social Work Public Health. 2016;31(4):215-30.
44. Kolk A, Lenfant F. Business–NGO collaboration in a conflict setting: partnership activities in the Democratic Republic of Congo. Business and Society. 2012;51(3):478-511.
45. Kotsiou OS, Kotsios P, Srivastava DS, Kotsios V, Gourgoulianis KI, Exadaktylos AK. Impact of the refugee crisis on the Greek healthcare system: a long road to Ithaca. Int J Environ Res Public Health. 2018;15(8).
46. Lacey G. Delivering culturally sensitive, sexual health education in western Kenya: a phenomenological case study. Afr J AIDS Res. 2017;16(3):193-202.
47. Landegger J, Hau M, Kaducu F, Sondorp E, Mayhew S, Roberts B. Strengths and weaknesses of the humanitarian Cluster Approach in relation to sexual and reproductive health services in northern Uganda. International Health. 2011;3(2):108-14.
48. Leisinger KM, Garabedian LF, Wagner AK. Improving access to medicines in low and middle income countries: Corporate responsibilities in context. Southern Medical Review. 2012;5(2):3-8.
49. Lepine A, Chandrashekar S, Shetty G, Vickerman P, Bradley J, Alary M, Moses S, CHARME India Group, Vassall A. What determines HIV prevention costs at scale? Evidence from the Avahan programme in India. Health Econ. 2016;25 Suppl 1:67-82.
50. Lépine, A, Vassall, A, Chandrashekar, S, Blanc, E, Le Nestour, A. Estimating unbiased economies of scale of HIV prevention projects: A case study of Avahan. Social Science and Medicine. 2015;131:164-172.
51. LoGiudice DC, Smith K, Shadforth G, Lindeman M, Carroll E, Atkinson D, Schaper F, Lautenschlager N, Murphy R, Flicker L. Lungurra Ngoora - a pilot model of care for aged and disabled in a remote Aboriginal community - can it work? Rural Remote Health. 2012;12:2078.
52. Mackintosh M, Chaudhuri S, Mujinja PG. Can NGOs regulate medicines markets? Social enterprise in wholesaling, and access to essential medicines. Global Health. 2011; 28(7):4.
53. Maher EJ. Managing the consequences of cancer treatment and the English National Cancer Survivorship Initiative. Acta Oncol. 2013;52(2):225-32.
54. Martiniuk AL, Adunuri, N, Negin, J, Tracey, P, Fontecha, C, Caldwell, P. Primary care provision by volunteer medical brigades in Honduras: a health record review of more than 2,500 patients over three years. International Journal of Health Services: planning, administration, evaluation. 2012;42(4):739-53.
55. Mkoka DA, Kiwara, A, Goicolea, I, Hurtig, AK. Governing the implementation of Emergency Obstetric Care: experiences of Rural District Health Managers, Tanzania. BMC Health Services Research. 2014;14:333.
56. Momoh. GT, Oluwasanu MM, Oduola OL, Delano GE, Ladipo OA. Outcome of a reproductive health advocacy mentoring intervention for staff of selected non- governmental organisations in Nigeria. BMC Health Services Research. 2015;15(1):1-9.
57. Neil AL, Carr VJ, Mihalopoulos C, Mackinnon A, Morgan V. Costs of psychosis in 2010: findings from the second Australian National Survey of Psychosis. Australian and New Zealand Journal of Psychiatry. 2014;48(2):169-182.
58. Ngo AD, Hill PS. The use of reproductive healthcare at commune health stations in a changing health system in Vietnam. BMC Health Services Research. 2011;11:237
59. Nurse K, Wight D. Development assistance and research capacity strengthening: The commissioning of health research in East Africa. Journal of Eastern African Studies. 2011; 5(2):233-251.
60. Nyambe A, Kampen JK, Baboo SK, Van Hal G. The impact of the social environment on Zambian cervical cancer prevention practices. BMC Cancer. 2018;18.
61. Olobo-Okao J, Sagaki P. Leishmaniasis in Uganda: Historical account and a review of the literature. Pan Afr Med J. 182014.
62. Ott MA, Rouse M, Resseguie J, Smith H, Woodcox S. Community-level successes and challenges to implementing adolescent sex education programs. Matern Child Health J. 2011;15(2):169-77.
63. Patel P. Forced sterilization of women as discrimination. Public Health Reviews. 2017;38(1):1-12.
64. Paz-Pacheco E, Jimeno C. Diabetes care in the Philippines. Journal of the Asean Federation of Endocrine Societies. 2015;30(2).
65. Price S. Professionalizing midwifery: exploring medically imagined labor rooms in rural Rajasthan. Medical Anthropology Quarterly. 2014;28(4):519-536.
66. Puett C, Guerrero S. Barriers to access for severe acute malnutrition treatment services in Pakistan and Ethiopia: a comparative qualitative analysis. Public Health Nutrition. 2015;18(10):1873-82.
67. Rutkow L, Jones-Smith J, Walters HJ, O'Hara M, Bleich SN. Factors that encourage and discourage policy-making to prevent childhood obesity: Experience in the United States. J Public Health Policy. 2016;37(4):514-27.
68. Sanghvi T, Haque R, Roy S, Afsana K, Seidel R, Islam S, Jimerson A, Baker J. Achieving behaviour change at scale: Alive & Thrive's infant and young child feeding programme in Bangladesh. Matern Child Nutr. 2016;12(Suppl 1):141-54.
69. Sarwar MR. Bangladesh health service delivery: innovative NGO and private sector partnerships. IDS Bulletin-Institute of Development Studies. 2015;46(3):17-28.
70. Simpson A, Bond V. Narratives of nationhood and HIV/AIDS: reflections on multidisciplinary research on the HIV/AIDS epidemic in Zambia over the last 30 years. Journal of Southern African Studies. 2014;40(5):1065-1089.
71. Song P. The Ice Bucket Challenge: The public sector should get ready to promptly promote the sustained development of a system of medical care for and research into rare diseases. Intractable Rare Dis Res. 2014;3(3):94-6.
72. Sparks M. Governance beyond governments: the role of NGOs in the implementation of the FCTC. Global health promotion. 2010;17(Suppl. 1):76-72.
73. Stark D, Lewis I. Improving outcomes for teenagers and young adults (TYA) with cancer. Klin Padiatr. 2013;225(6):331-4.
74. Suplee JB. The ACA and the undocumented. The American Journal of Nursing. 2012;112(4):21-7.
75. Tabash T, Abuqamar M. Assessment of the status of private and non-governmental pharmaceuticals supply warehouses in Gaza Strip, Palestine. Asian Journal of Pharmaceutical and Clinical Research. 2018;11(2):300-7.
76. Thomson DR, Hadley MB, Greenough PG, Castro MC. Modelling strategic interventions in a population with a total fertility rate of 8.3: A cross-sectional study of Idjwi Island, DRC. BMC Public Health. 2012;12:12.
77. Tomlinson E, Spector A, Nurock S, Stott J. Euthanasia and physician-assisted suicide in dementia: A qualitative study of the views of former dementia carers. Palliat Med. 2015;29(8):720-6.
78. Tsolekile LP, Schneider H, Puoane T. The roles, training and knowledge of community health workers about diabetes and hypertension in Khayelitsha, Cape Town. Curationis. 2018;41(1):e1-e8.
79. Wallington S, Oppong B, Dash C, Coleman T, Greenwald H, Torres T, Iddirisu M, Adams-Campbell LL. A community-based outreach navigator approach to establishing partnerships for a safety net mammography screening center. J Cancer Educ. 2018;33(4):782-7.
80. Walsh A, Mulambia C, Brugha R, Hanefeld J. "The problem is ours, it is not CRAIDS'". Evaluating sustainability of community based organisations for HIV/AIDS in a rural district in Zambia. Globalization and Health. 2012;28(8):40
81. Waters HC, Davidson S. “A unique little microcosm”: exploring a self‐sustaining community project which harnessed social action in a public space - Waters - 2018 - Journal of Community Psychology - Wiley Online Library. Community Psychology. 2018;46(8):1045-61.
82. Zaidi S, Gul X, Nishtar NA. Parallel NGO networks for HIV control: risks and opportunities for NGO contracting. Global journal of health science. 2013;5(2):171-175.
